# Regionally Distinct Immune and Metabolic Transcriptional Responses in the Bovine Small Intestine and Draining Lymph Nodes During a Subclinical *Mycobacterium avium* subsp. *paratuberculosis* Infection

**DOI:** 10.3389/fimmu.2021.760931

**Published:** 2021-12-15

**Authors:** Eveline M. Ibeagha-Awemu, Nathalie Bissonnette, Duy N. Do, Pier-Luc Dudemaine, Mengqi Wang, Antonio Facciuolo, Philip Griebel

**Affiliations:** ^1^ Sherbrooke Research and Development Centre, Agriculture and Agri-Food Canada, Sherbrooke, QC, Canada; ^2^ Vaccine and Infectious Disease Organization (VIDO), University of Saskatchewan, Saskatoon, SK, Canada; ^3^ School of Public Health, University of Saskatchewan, Saskatoon, SK, Canada

**Keywords:** *Mycobacterium avium* subsp. paratuberculosis, bovine small intestine (jejunum/ileum/ileum lymph node/jejunum lymph node), *CD14/LOC616364/ENSBTAG00000027033*, Hox genes, phagosome pathway/IL-17 signaling pathway/toll-like receptor signaling pathway/NF-κB signaling pathway/chemokine signalling pathway/HIF signaling pathway/fat digestion and absorption pathway

## Abstract

*Mycobacterium avium* subsp. *paratuberculosis* (MAP) is the causative infectious agent of Johne’s disease (JD), an incurable granulomatous enteritis affecting domestic livestock and other ruminants around the world. Chronic MAP infections usually begin in calves with MAP uptake by Peyer’s patches (PP) located in the jejunum (JE) and ileum (IL). Determining host responses at these intestinal sites can provide a more complete understanding of how MAP manipulates the local microenvironment to support its long-term survival. We selected naturally infected (MAPinf, n=4) and naive (MAPneg, n=3) cows and transcriptionally profiled the JE and IL regions of the small intestine and draining mesenteric lymph nodes (LN). Differentially expressed (DE) genes associated with MAP infection were identified in the IL (585), JE (218), jejunum lymph node (JELN) (205), and ileum lymph node (ILLN) (117). Three DE genes (*CD14*, *LOC616364* and *ENSBTAG00000027033*) were common to all MAPinf *versus* MAPneg tissues. Functional enrichment analysis revealed immune/disease related biological processes gene ontology (GO) terms and pathways predominated in IL tissue, indicative of an activated immune response state. Enriched GO terms and pathways in JE revealed a distinct set of host responses from those detected in IL. Regional differences were also identified between the mesenteric LNs draining each intestinal site. More down-regulated genes (52%) and fewer immune/disease pathways (n=5) were found in the ILLN compared to a higher number of up-regulated DE genes (56%) and enriched immune/disease pathways (n=13) in the JELN. Immunohistochemical staining validated myeloid cell transcriptional changes with increased CD172-positive myeloid cells in IL and JE tissues and draining LNs of MAPinf *versus* MAPneg cows. Several genes, GO terms, and pathways related to metabolism were significantly DE in IL and JE, but to a lesser extent (comparatively fewer enriched metabolic GO terms and pathways) in JELN suggesting distinct regional metabolic changes in IL compared to JE and JELN in response to MAP infection. These unique tissue- and regional-specific differences provides novel insight into the dichotomy in host responses to MAP infection that occur throughout the small intestine and mesenteric LN of chronically MAP infected cows.

## Introduction

*Mycobacterium avium* subsp. *paratuberculosis* (MAP) is the causative agent of Johne’s disease (JD) in ruminants (1, 2). JD imposes a substantial economic burden on dairy and beef industries worldwide (1, 3, 4). JD and MAP has attracted further attention because of a possible connection to human Crohn’s disease (CD) (5, 6). Following MAP infection, JD progresses slowly and can be divided into three disease stages including silent infection, subclinical, and clinical/advanced disease (7). MAP infection occurs by fecal-oral transmission or by calves ingesting colostrum/milk containing MAP. MAP breaches the epithelial barrier of the small intestine predominantly *via* microfold cells in Peyer’s patches (PP), and is taken up by subepithelial macrophages where it effectively subverts host cell responses to promote intracellular survival and replication (7, 8). Bacterial evasion of host cell responses involves numerous mechanisms, including suppression of apoptotic processes, inhibition of antigen presentation, and ultimately blocking phago-lysosomal maturation and fusion to facilitate MAP survival in phagosomes (9, 10). The outcome of MAP infection is dependent on the interaction between infected cells (e.g. macrophages) and lymphoid cells, specifically T-cells which mediate a protective response that either contains or controls MAP infection. Progression to clinical disease is thought to occur when animals fail to contain MAP infection or the host immune response shifts from a cell-mediated response to a non-protective humoral response (11).

Several transcriptome and gene expression studies have described host cell responses to MAP infection in different cell types (e.g. macrophage, leukocyte), blood, and intestinal tissues (12–20). The bovine small intestine, the initial site of MAP infection, is characterized by regional differences in mucosa-associated lymphoid tissues that vary in both morphology and function. Recently, Facciuolo et al. (20) identified regional differences in the responses of mucosa-associated lymphoid tissues (i.e. PP) following experimental MAP infection of neonatal calves. However, it remains to be determined whether intestinal tissue beyond the boundaries of organized lymphoid tissues, such as PP, develop differential responses to MAP infection. Regional differences have been identified in the abundance of myeloid and dendritic cell populations located in the intestinal lamina propria (LP) of young calves (21). Therefore, we postulated that regional differences in intestinal tissue, similar to that observed in the PPs, could significantly affect host response to MAP infection in the intestinal LP. Additionally, it is not clear how the mesenteric lymph nodes draining these distinct anatomical sites respond to MAP infection. Knowledge of the transcriptome at sites of enteric infection will serve to enhance our understanding of MAP pathogenesis and support rational design of vaccines and/or antimycobacterial therapeutic modalities. Therefore, in the current study, we combined RNA sequencing analyses, gene ontology/pathways approaches and immunohistochemical (IHC) staining to gain novel insight into regional host responses to persistent MAP infection within the small intestine, including the ileum (IL), ileal lymph node (ILLN), jejunum (JE) and jejunal lymph node (JELN) of cows with a subclinical, persistent MAP infection.

## Material and Methods

### Animal Selection and JD Diagnosis

Animals selected for this study were from companion research studies (19, 22, 23) which includes 16 commercial dairy farms (tie and free stall) with previously diagnosed JD cases in the province of Québec, Canada. Briefly, cows were at least 24 months old at first sampling and paired blood and faecal samples (collected at the same time) were tested every six months over a one to four year period, as described (23). Blood and feces were respectively tested for the presence of MAP-specific antibodies using the Pourquier ELISA assay (IDEXX Laboratories, Markham, Ontario, Canada) and by fecal quantitative PCR as described (22), following the manufacturer’s instructions. The number of MAP particles excreted in feces was evaluated using standard curves of 5 different strains made with 9 serial dilutions (5 and 2 fold, alternatively) from 10 pg to 0.001 pg of purified MAP genomic DNA representing 2000 to 0.2 genomic copies, respectively, based on a genome size of 4.83Mb. The standard curves were used to extrapolate gene copy number in one gram of feces. Feces from the cows selected for the present study were also tested to confirm live MAP excretion by the mycobacterial culture method at the Laboratoire d’épidémiosurveillance animale du Québec (Saint-Hyacinthe, Québec, Canada) as described previously (24). Cows were designated negative [MAPneg] if both tests (serum ELISA and fecal culture) were negative (−/−; *n* = 5) or positive [MAPinf] if both tests were positive (+/+; *n* = 4) (**Table 1**). Animals selected for the collection of IL, ILLN, JE and JELN tissues were humanely euthanized by intra-venous administration of 5 mg detomidin and 120 mL euthansol. Jejunal tissues without visible Peyer’s patches (PP) were collected at the mid-jejunum and JELN tissues were collected proximal to this site. Ileum tissues (without visible PP) were collected at ~35cm proximal to the ileocecal valve and mesenteric lymph nodes were also collected adjacent to this site. Tissues were immediately snap frozen in liquid nitrogen and stored at -80°C until RNA isolation. To confirm that collected tissues were infected with MAP, the tissues were tested by real time quantitative PCR (qPCR).

**Table 1:** MAP infectious status of cows and analyzed tissues based on the results of three tests.

Cow ID	Age (yr/mth)^a^	Phenotype of cows^b^ (quantity of MAP in feces)	*f57* qPCR^c^/Acid fast staining of intestinal tissues			
			Jejunum	Jejunal LN	Ileum	Ileal LN
MAP-infected cows						
5678	3/5 - 4/0	High Shedder: F+/E+ ${}^{\left(1.05\text{ x }10^{6}/\text{gr feces}\right)}$	4.94 x 10^5^ ^AF+ (very sparse)^	2.76 x 10^6^ ^AF+ (very sparse)^	3.04 x 10^5^ ^AF+ (very sparse)^	5.38 x 10^5^ ^AF+ (very sparse)^
8156	2/4 - 3/3	High Shedder: F+/E+ ${}^{\left(1.55\text{ x }10^{4}/\text{g feces}\right)}$	N/AF-	3.79 x 10^5^ ^AF-^	7.24 x 10^4^ ^AF-^	2.49 x 10^5^ ^AF+ (very sparse)^
1180	3/4	High Shedder: F+/E+ ${}^{\left(8.6\text{ x }10^{6}/\text{g feces}\right)}$	4.02 x 10^8^ ^AF+ (very abundant)^	5.80 x 10^7^ ^AF+ (abundant)^	1.32 x 10^8^ ^AF+ (very abundant)^	3.46 x 10^7^ ^AF+ (abundant)^
5144	5/6 - 7/3	High Shedder: F+/E+ ${}^{\left(1.55\text{ x }10^{4}/\text{g feces}\right)}$	1.95 x 10^4^ AF-	N AF-	2.64 x 10^4^ AF-	N AF-
MAP negative cows						
8664	4/2 - 5/1	Negative: F-/E- ^(Not detected)^	N AF-	N AF-	N AF-	N AF-
1376	5/5 - 7/7	Negative: F-/E- ^(Not detected)^	N AF-	N AF-	N AF-	N AF-
3403	4/9 – 6/10	Negative: F-/E- ^(Not detected)^	N AF-	N AF-	N AF-	N AF-
6528^d^	1/7 - 4/2	Potentially tolerant: F-/E- ^(Not detected)^	N AF-	N AF-	3.44 x 10^4^ AF-	N AF-
117^d^	5/9 - 7/5	Potentially tolerant: F- and +/E- ^(Not detected)^	N AF-	N AF-	3.13 x 10^6^ AF-	N AF-

a Age of animal at start and end of testing. Animals were tested twice a year.

b The phenotype of cows was deduced from the results of two tests (fecal qPCR using MAP specific ISMap02 target (F) and Pourquier serum ELISA assay (E)) over a period of 1 to 4 years, with two tests each per year. The presence of live MAP was confirmed in fecal samples from four MAP-infected cows and were not detected in MAP negative cows.

c f57 gene copy number per gram of tissue; N, PCR-negative; LN, lymph node; AF, Acid fast.

d Cows were not included in the transcriptome analyses due to presence of MAP in the sampled ileal tissues and or due to low but detectable intermittent (once or twice) MAP fecal excretion during the period of testing. For cow 6528, both fecal qPCR and ELISA results were below detection limits throughout the duration of the study.

### *F57* Real Time qPCR

Quantification of MAP bacterial burden in tissue samples was determined using real-time qPCR targeting the MAP-specific, single-copy DNA element *f57* (25). Real time qPCR reactions were performed in triplicate, containing PerfeCTa SYBR Green SuperMix (Quanta Biosciences, Inc. Beverly, MA, USA), 300 nM of *f57* primers (26), and 50 ng of extracted tissue DNA. Cycling conditions were initial denaturation for 3 min at 95°C followed by 36 cycles of 95°C for 15 s, 60°C for 30 s, and 72°C for 30 s using a Bio-Rad CFX Connect Real-Time PCR Detection System (Bio-Rad Laboratories, Inc. Mississauga, ON, Canada). Quantitative threshold cycle (Cq) for each reaction was determined by CFX Manager™ Software (Bio-Rad Laboratories), and average Cq calculated using arithmetic average of triplicate reactions. For each PCR plate, five 10-fold serial dilutions of purified MAP genomic DNA representing 1 × 10^6^ to 1 x 10^1^ genomic copies (60 ng to 6 ng, respectively, based on a genome size of 4.83Mb) was amplified to generate a standard curve from which to extrapolate gene copy number in each tissue sample. Data are expressed as *f57* gene copy number per gram of tissue (**Table 1**).

### Histology and Immunohistochemistry

Approximately 3-4 mm^2^ pieces of tissue were collected at the same sites sampled for gene expression analysis and tissues were immediately embedded in OCT (Optimal cutting temperature compound) and stored in sealed containers at -80°C. Tissue sections (8 μm) were cut for hematoxylin and eosin (H&E) staining, acid fast staining, and immunohistochemical staining at the Plateforme d’histologie et microscopie électronique, Faculté de médecine, Université de Sherbrooke.

For immunohistochemistry, the primary antibodies were polyclonal rabbit anti-human CD3 (clone A0452, 1:400 dilution) (DAKO, Glostrup, Denmark), anti-bovine CD11c mAb (clone BAQ153A, 1:50 dilution)] (Kingfisher Biotech, Saint Paul, MN, USA), mouse anti-bovine CD172a mAb (clone CC149, 1:500 dilution) (Bio-Rad Laboratories, Mississauga, ON, Canada), mouse anti-Ki67 (clone MIB-1, 1:50 dilution) (Agilent Technologies, Mississauga, ON, Canada), and a purified mouse IgG1 isotype control (product code MG100, 1:50 dilution) (Life Technologies, by Thermo Fisher Scientific, Mississauga, ON, Canada). Histological images of the stained tissues were acquired using a slide scanner (NanoZoomer Digital Pathology, Hamamatsu Photonics, Boston, MA, USA) followed by viewing with NDP.view2 viewing software U12388-01 (Hamamatsu Photonics, Boston, MA, USA).

### RNA Isolation

Total RNA was extracted from tissue samples using miRNeasy Kit (Qiagen Inc., Toronto, ON, Canada). The tissues (30 mg/sample) were homogenized in 700 µL TRIzol Reagent (Life Technologies) using a Polytron homogenizer (Polytron PT 10-35 GT, Kinematica AG, Luzern, Switzerland) with a 7 mm probe for 10 s at 12,000 rpm. This step was repeated three times with incubation on ice between repetitions. Following 5 min incubation at room temperature, 140 µL chloroform was added to the mixture and vortexed vigorously for 20 s. The mixture was then centrifuged (15 min at 12,000 × g at 4°C) followed by addition of 1.5 volumes ethanol (100%). The aqueous phase was transferred to a column and wash steps performed according to manufacturer’s recommendations. RNA was eluted twice using 30 µL nuclease-free water each time. Total RNA (10 µg) was subjected to DNase treatment using Turbo DNA-free™ Kit (Ambion Inc. Foster City, CA, USA) to remove any contaminating DNA. Nanodrop ND-1000 (NanoDrop Technologies, Wilmington, DE, USA) was used to determine RNA concentration before and after DNase treatment and RNA integrity was assessed with an Agilent 2100 Bioanalyzer (Agilent Technologies, Santa Clara, CA, USA) using the RNA 6000 Nano Labchip Kit (Agilent Technologies) after DNase treatment. All samples had a RNA integrity number (RIN) value ≥7.0.

### Library Construction and Sequencing

Ribosomal RNA (rRNA) depletion was conducted with 4 µg of total RNA from each sample using Ribo-Zero Gold rRNA Removal Kit (Illumina Inc., San Diego, CA, USA) and following the manufacturer’s recommendations. Libraries for sequencing were prepared from rRNA depleted RNA (2.5 µg/sample) using NEBNext Ultra Directional RNA Library Prep Kit for Illumina (New England Biolabs, Whitby, ON, Canada) and barcoded with NEBNext Multiplex Oligos for Illumina^®^ (New England Biolabs) to facilitate multiplexing. A Quant-iT PicoGreen double-stranded DNA (dsDNA) Assay Kit (Life Technologies) was used to measure the final concentration of each library. Insert size of libraries was estimated using High Sensitivity DNA Analysis Kit (Agilent Technologies) with an Agilent 2100 Bioanalyzer (Agilent Technologies). Equimolar amounts of samples were pooled (n = 4 or 3 per lane) and paired-end sequenced (2 × 126 bp) on a High Throughput Model flow cell on an Illumina HiSeq 2500 system at The Centre for Applied Genomics, The Hospital for Sick Children, Toronto (http://www.tcag.ca/).

### Bioinformatics Processing of Data

Bioinformatics processing of generated RNA sequences was accomplished with a RNA-Seq pipeline version 2.2.0 developed by the bioinformatics team of McGill University and Genome Quebec Innovation Centre (MUGQIC), Montreal, Canada. Briefly, alignment of clean reads to the *Bos taurus* reference genome ARS-UCD1.2 was accomplished with STAR version 2.5.0c. Following the read count step, a threshold was applied to keep genes having at least one FPKM (Fragments Per Kilobase of transcript per Million mapped reads) in at least 50% of the libraries before proceeding with differential mRNA expression analysis.

### Differential mRNA Expression Analysis

The R (v3.1.3) package, Deseq2 (v1.11.19) (27) which uses a negative binomial model was used to identify significantly differentially expressed (DE) mRNAs when comparing MAP positive and negative cows for each tissue sampled. Significantly DE mRNAs were defined as having a Benjamini and Hochberg (28) corrected false discovery rate (FDR) p-value ≤ 0.05 and were used for gene ontology (GO) and pathways analyses, and analysis of protein-protein interactions.

### Gene Ontology and Pathways Enrichment

Functional enrichment of DE genes was performed using ClueGO program (http://apps.cytoscape.org/apps/cluego) (29). The *p*-value for enriched terms were adjusted with Benjamini-Hochberg correction of false discovery rate (FDR) (28). GO terms and Kyoto Encyclopedia of Genes and Genomes (KEGG) pathways were considered significantly enriched at FDR < 0.05.

### Protein Interaction Network

Potential interactions between DE genes were evaluated with the protein-protein interaction (PPI) network using STRING v10.5 (https://string-db.org). String uses eight major sources of interaction/association data, including known interactions (experimentally determined and curated database), predicted interactions (neighborhood, fusion and co-occurrence) and others (co-expression, protein homology and text mining) to define interactions between proteins using a probabilistic confidence score (30, 31). The combined score of all available resources were used to estimate the interaction strength between proteins.

### Quantification of Select DE Genes by Real-Time qPCR

Real time qPCR analysis was performed to confirm expression levels of 12 genes (8 DE and 4 non-DE) (**Supplementary Table S1**). Aliquots of total RNA (1 µg) from the same RNA used in RNA-Seq was reverse transcribed into cDNA using Invitrogen™ SuperScript™ III Reverse Transcriptase (Invitrogen, by Thermo Fisher Scientific, Mississauga, ON, Canada) and Invitrogen™ Random Primers (Invitrogen) according to manufacturer’s recommendations. Primers for gene specific amplification were designed with Integrated DNA Technologies RealTime qPCR Assay tool (https://www.idtdna.com/scitools/Applications/RealTimePCR/). Primer specificity was tested by confirming a single product of the expected size on 1.5% agarose gel. PCR efficiency for all primer pairs were 100% ± 10%. The cDNA was diluted to 20 ng/µL prior to gene specific qPCR amplification in triplicates using Applied Biosystems™ Power SYBR^®^ Green PCR Master Mix (Applied Biosystems, by Thermo Fisher Scientific, Mississauga, ON, Canada) as recommended by manufacturer. The qPCR reaction mix included 3 µL of cDNA, forward and reverse primers at 150 to 900 nM (**Supplementary Table S1**) and 5 µL of master mix. The amplification was performed using a StepOnePlus™ Real-Time PCR System (Applied Biosystems). The cycling condition was 10 min at 95°C for initial denaturation/activation followed by 40 cycles of 95°C for 30 s, 60°C for 30 s and 72°C for 30 s. *RPS15* and *ATP5B* were used as housekeeping genes to normalise the expression of genes. Relative quantification was done using 2^-ΔΔCt^ method (32).

## Results

### Identifying Naturally MAP-Infected Cows

Cows identified naturally infected with MAP (MAPinf) and age-matched tested-negative controls (MAPneg) were selected from a cohort of adult cows from 16 commercial dairy herds as part of a companion project (23). Animals identified as MAPinf or MAPneg were serially tested during the longitudinal study to identify possible uninfected cows and those with persistent MAP infection. As previously described (22), cows were considered to excrete MAP in their feces if the qPCR threshold cycle (Ct) was 38 or less, and non-shedders if Ct was 45; total amount of cycles was settled at 45. Regarding the detection of MAP-specific serum IgG antibodies, the threshold of 45 (sample/positive standard, S/P) was used. S/P higher than 45 is considered positive according to the manufacturer. According to these recommendations, five cows were phenotyped MAPneg. However, cows 6528 and 117 phenotyped MAPneg, presented S/P between 15 and 30 (cow 6528) during one isolated period or CT value of 36-38 (cow 117) during one isolated period, were considered to be of uncertain status. Four MAPinf cows confirmed excreting MAP using the MAP specific ISMap02 target, and with concordant serum ELISA results for the two consecutive sampling periods per year were selected for intestinal tissues analysis. Intestinal tissues and lymph nodes were collected from the four MAPinf cows and also from the five MAPneg cows.

To ensure that MAP was present in the collected tissues of the four MAPinf cows or absent in the tissues of the five MAPneg cows selected for further analysis, qPCR was performed to detect the *f57* single-copy DNA element unique to MAP (25). The *f57* qPCR analysis detected MAP in tissues collected from the four cows phenotyped as MAPinf while MAP was absent in all tissues from three cows phenotyped as MAPneg. However, MAP was detected in IL tissue samples collected from two cows (117 and 6528) initially phenotyped as MAPneg according to the manufacturer’s recommendation but were considered suspicious for several reasons (**Table 1**). First, out of the two suspicious cows, cow 6528 was found to excrete once very low amount of MAP (Ct = 36-38). Meanwhile, the S/P results from serum ELISA analysis of cow 117 during one isolated period oscillated between 15 and 30 during the longitudinal study. Although they were not qualified for MAPinf group because MAP-specific serum IgG antibodies were below the threshold level to consider positivity to JD, according to the company’s criteria; the presence of MAP was established by *f57* qPCR in ileal intestinal tissues (only) (**Table 1**), and were removed from the MAPneg group. This result confirms that cows with intermittent MAP excretion in their faeces and with serum ELISA values oscillating above the threshold should not be considered MAP free. Moreover, the results of the three tests (fecal, serum and *f57* qPCR) for cows 6528 and 117 fit the MAP tolerant phenotype described in our recent manuscript (19).

Acid-fast staining of tissue sections were consistent with the results of *f57* qPCR. No acid-fast bacteria were observed in the MAPneg samples but highly abundant acid-fast bacteria were observed in tissues from one MAPinf cow (#1180) with a high MAP copy number and abundant acid-fast bacteria were observed in tissue sections from cows with a lower MAP copy number (**Table 1**).

### Mapping Statistics, Gene Expression Profiles, and Differentially Expressed Genes

A total of 551 million (M), 561 M, 562 M and 553 M reads were generated for IL, ILLN, JE and JELN tissues, respectively. After trimming adaptors and removal of low quality reads, 94.6%, 95.5%, 94.9% and 95.5% of total reads were mapped to the bovine genome for IL, ILLN, JE and JELN, respectively (**Supplementary Table S2**). The rate of uniquely mapped reads compared to mapped reads was 74.6%, 75.7%, 71.6% and 75.1% for IL, ILLN, JE and JELN, respectively. A total of 17,443, 16,787, 17,501, and 16,713 genes [considering read counts per million mapped reads (CPM) > 1] were detected in the IL, ILLN, JE and JELN, respectively. In these tissues, altogether, 15,643 genes were identified. Jejunum had the highest number of unique genes (128) while the lowest number of unique genes (33) was observed in the ILLN.

**Figure 1:**
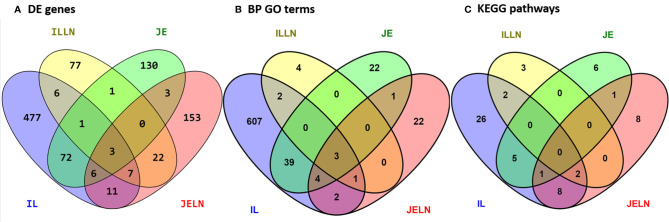
Number of unique and shared differentially expressed genes **(A)**, enriched biological process gene ontology terms **(B)**, and pathways **(C)** between MAP infected and MAP negative ileum (IL), ileum lymph node (ILLN), jejunum (JE) and jejunum lymph node (JELN) tissues.

To determine if subclinical MAP infection results in regional- and tissue-specific host responses in the small intestine, we utilized RNA-Seq to compare the transcriptomes of jejunal and ileal intestinal tissue and associated draining mesenteric lymph nodes from MAPinf cows (n=4; **Table 1**) *versus* MAPneg cows (n=3; **Table 1**). In IL, ILLN, JE and JELN tissues, a total of 585, 117, 218 and 205 genes, respectively, were significantly DE (FDR < 0.05) when comparing MAPinf *versus* MAPneg cows (**Figure 1A** and **Supplementary Tables S3A–D**). Three DE genes (*CD14*, *ENSBTAG00000027033* and *LOC616364* [C-C motif chemokine 3]) were common to all tissues. IL and JE shared the highest number of common DE genes (82) while both lymph node tissues shared fewer DE genes; 32 were common for ILLN and JELN (**Figure 1A**). The tissues with its associated lymph node had few common DE genes: IL and ILLN (17) and JE and JELN (12) (**Figure 1A**).

Analysis of IL tissues revealed 585 DE genes (*FDR* < 0.05) among which 154 genes were down-regulated and 431 genes were up-regulated (**Supplementary Table S3A**). Among the 218 DE genes from the JE tissues, 83 and 135 genes were down- and up-regulated, respectively (**Supplementary Table S3B**). The top five DE genes with the lowest FDR or DE genes with >4 log2 fold change (L2FC) between MAPinf and MAPneg cows in the IL or JE are shown in **Table 2**. Notable significant DE genes included *TLR4* (FDR=4.70E-18), *DUOX2* (FDR=2.55E-13), *ENSBTAG00000048135* (FDR=1.14E-12), *GAL3ST1* (FDR=1.29E-09), and *CD14* (FDR=9.57E-09). In the IL, and *ENSBTAG00000027033* (FDR=3.03E-12), *CD163* (FDR=6.53E-10), *CTGF* (FDR=3.75E-07), *ENSBTAG00000037537* (FDR=5.04E-07) and *ENSBTAG00000039446* (FDR=1.30E-06) in the JE (**Table 2** and **Supplementary Tables S3A, B**). Moreover, numerous myeloid genes, notably the macrophage antigen *CD68* and dendritic cell antigen *DCSTAMP*, and other genes with diverse functions (e.g. *CD68*, *DCSTAMP, S100A12, S100A8, S100A9, CLEC4E*, and *CXCL9*) were changed at > 4 L2FC in IL and JE or in IL or JE only (**Table 2**).

**Table 2:** Select significantly differentially expressed (DE) genes (top 5 DE genes based on adjusted *p*-values or genes with log2fold change >4) in ileum and jejunum tissues of MAP-infected cows^1^.

Gene Symbol^2^	L2FC^3^	FDR^4^	Gene Symbol	L2FC	FDR^4^
**Ileum**					
TLR4	1.2269	4.70E-14	ENSBTAG00000001595	4.128	0.007548
DUOX2	7.073	2.55E-13	F11	4.116	0.019997
ENSBTAG00000048135	3.3546	1.14E-12	PTX3	4.097	0.003648
GAL3ST1	-5.017	1.29E-09	TM4SF19	4.080	0.00011
CD14	2.8511	9.57E-09	TNIP3	4.047	0.008528
IRG1	5.019	8.52E-08	ENSBTAG00000021407	-4.061	0.022531
SLC13A5	6.762	1.12E-07	CCDC180	-4.099	0.000145
MX2	4.026	4.49E-07	LOC538060	-4.317	0.044179
CLEC4E	8.725	7.24E-07	CFAP221	-4.715	0.003069
PLA2G5	5.44	7.29E-07			
DUOXA2	5.658	2.36E-06	**Jejunum**		
CD68	4.385	6.22E-06	ENSBTAG00000027033	-2.935	3.03E-12
ENSBTAG00000046901	5.426	6.60E-06	CD163	3.729	6.53E-10
ENSBTAG00000010433	7.903	9.15E-06	CTGF	2.661	3.75E-07
S100A12	7.758	1.11E-05	LOC618463	-2.94	5.04E-07
CXCL9	5.176	1.35E-05	LOC100850808	-4.858	1.30E-06
DCSTAMP	8.502	1.52E-05	PLA2G2A	-4.11	3.54E-05
S100A8	7.937	2.61E-05	A4GNT	-4.218	6.92E-05
TAP	5.00	3.68E-05	S100A12	7.754	0.000107
ENSBTAG00000027225	9.929	0.002833	SLC13A5	5.853	0.00045
ENSBTAG00000047284	9.17	0.000663	OLR1	5.285	0.000597
ENSBTAG00000018189	7.152	0.00011	IRG1	4.699	0.000597
BPIFB1	6.269	0.034515	PLA2G5	5.151	0.000845
HP	5.756	0.018674	DCSTAMP	10.27	0.002456
PFKFB1	5.725	0.016039	CLEC4E	8.875	0.001873
GPNMB	5.465	0.006815	ENSBTAG00000047284	8.676	0.001478
ENSBTAG00000047363	5.396	0.011158	ENSBTAG00000018189	7.143	0.002015
SLC36A2	5.37	0.026754	S100A8	5.783	0.001942
S100A9	5.339	0.00493	GPNMB	5.745	0.041286
CLEC4F	5.266	0.003803	LYZ	5.048	0.001604
LYZ	4.87	0.004746	CD1E	4.974	0.002644
CD1E	4.858	0.001136	TXLNB	4.586	0.016286
EMR1	4.612	0.000172	S100A9	4.367	0.035449
OLR1	4.535	0.002004	CD68	4.359	0.002644
CYP27B1	4.512	0.00456	DUOX2	4.108	0.02088
LOC616364	4.456	0.010438	SLC31A2	4.095	0.01339
LOC281376	4.453	0.049814	TRPV1	4.043	0.001262
SDS	4.27	0.005528	MPTX	-5.133	0.021658
NOS2	4.198	0.00095	ST6GALNAC1	-5.264	0.004128
LF	4.156	0.002522	LOC508916	-6.653	0.002644
SLC11A1	4.149	0.005528			

^1^See **Supplementary Tables 3A, B** for complete DE results. ^2^ENSBTAG and LOC notations are used for novel uncharacterized genes only. ^3^L2FC, log2fold change. ^4^FDR = Adjusted p-values with Benjamini-Hochberg correction for false discovery rate.

Transcriptional profiling of the mesenteric lymph nodes revealed 117 DE genes in the ILLN and 205 DE genes in the JELN of MAPinf cows when compared to MAPneg cows (**Supplementary Tables S3C, D**). The most significant DE genes included *ADCYAP1R1* (FDR = 1.81E-06), *ENSBTAG00000039041* (FDR=3.09E-06), *PFKFB3* (FDR=1.32E-05) in the ILLN, and *ENSBTAG00000037578* (FDR=1.00E-08), *FAM234B* (FDR=1.09E-08) and *TTC21B* (FDR=1.51E-06) in the JELN (**Table 3**). Interestingly, more of the DE genes were down-regulated in the ILLN (52%) compared to JELN (42%). Some DE genes with important L2FC (>3) and having roles in the immune function or metabolism were detected in the ILLN and or JELN (e.g. *IGHE, DCSTAMP, CLEC4E*, *FABP1*, *STAR* and *DPEP1*) (**Table 3**). It is noteworthy that an appreciable number of novel and uncharacterized genes were highly DE in the analysed tissues (**Tables 2**, **3**, and **Supplementary Tables S3A‒D**).

**Table 3:** Select significantly differentially expressed (DE) genes (top 8 DE genes based on adjusted p-values or genes with log2fold change ≥2.5 (ileum lymph node) or >3 (jejunum lymph node) of MAP-infected cows^1^.

Gene symbol	L2FC^2^	P-value	FDR^3^	Gene symbol	L2FC	P-value	FDR
Ileum lymph node				Jejunum lymph node			
ADCYAP1R1	-1.1501	1.24E-10	1.81E-06	ENSBTAG00000037578	-4.9289	6.63E-13	1.00E-08
ENSBTAG00000039041^4^	-2.1806	4.24E-10	3.09E-06	FAM234B	1.4093	1.44E-12	1.09E-08
PFKFB3	1.3592	2.72E-09	1.32E-05	TTC21B	-0.8649	2.99E-11	1.51E-07
ENSBTAG00000037578	-4.5415	7.58E-09	1.84E-05	GLDN	3.4804	8.52E-10	3.22E-06
IFITM10	-2.0209	6.73E-09	1.84E-05	ENSBTAG00000027033	-4.8037	3.16E-09	7.98E-06
SLC17A9	1.2644	6.73E-09	1.84E-05	EYA3	0.9717	2.75E-09	7.98E-06
CCL2	-2.3597	1.40E-08	2.92E-05	TMEM189	1.2637	6.43E-09	1.39E-05
ENSBTAG00000048030	2.5035	2.19E-08	4.00E-05	HRSP12	1.5378	3.68E-08	6.97E-05
ENSBTAG00000038067	4.358473	1.35E-05	0.005941	DCSTAMP	7.4312	0.000671	0.049764
IGHE	4.03509	0.000221	0.038796	FABP1	5.9846	0.000292	0.029501
IGFBP2	3.309499	1.72E-07	0.000232	STAR	5.6718	0.000503	0.042729
LOC616364	2.682174	2.84E-05	0.009611	PRKAG3	5.191	0.000502	0.042729
ENSBTAG00000048030	2.503529	2.19E-08	4.00E-05	CLEC4E	4.5716	0.000484	0.041929
QPCT	-2.5000	5.51E-07	0.000501	CYP27B1	4.3899	0.000159	0.020506
SLC16A12	-2.51287	0.000149	0.029818	LOC515676	4.186	1.19E-05	0.004202
SLC45A3	-2.53294	0.000302	0.043831	ENSBTAG00000030517	3.8552	0.000594	0.046625
PTN	-2.76337	3.15E-05	0.010444	KCNMA1	3.6756	0.000457	0.041418
RGS13	-2.81954	1.02E-05	0.00497	CPS1	3.5005	9.69E-05	0.01481
AK5	-3.01336	0.000285	0.043831	ASGR2	3.4819	8.41E-06	0.003541
CPA3	-3.08712	1.67E-06	0.001222	GLDN	3.4804	8.52E-10	3.22E-06
SPTA1	-3.18683	0.000175	0.033582	C9	3.4511	0.000213	0.024343
ENSBTAG00000038565	-3.21279	0.000249	0.040873	MREG	3.2766	9.77E-06	0.003791
DDIT4L	-3.23912	2.68E-05	0.009516	ENSBTAG00000043572	3.2617	4.55E-05	0.009847
ENSBTAG00000035183	-3.24504	0.000359	0.046033	TPSB1	-3.032	1.52E-05	0.004434
ELMOD1	-3.35654	8.91E-05	0.020941	SLC14A1	-3.057	9.50E-05	0.01481
TPSB1	-3.4669	0.00042	0.050979	PTI	-3.455	8.13E-05	0.013984
PI16	-3.79254	4.81E-06	0.002921	LOC616039	-3.484	8.66E-08	0.000119
PTI	-3.87735	2.78E-07	0.00027	TPSB2	-3.634	3.12E-06	0.001888
LOC616039	-4.1055	2.30E-07	0.000258	SPTA1	-3.634	0.000214	0.024343
ENSBTAG00000037578	-4.54151	7.58E-09	1.84E-05	CYR61	-3.732	0.000112	0.016058
ENSBTAG00000027033	-4.79121	1.37E-07	0.000222	EBF2	-3.773	0.000652	0.048891
				NAALADL1	-4.469	2.36E-07	0.00021
				ENSBTAG00000027033	-4.804	3.16E-09	7.98E-06
				GRIN3A	-4.835	1.29E-05	0.004321
				ENSBTAG00000037578	-4.929	6.63E-13	1.00E-08
				DPEP1	-5.018	9.07E-05	0.014452

^1^See **Supplementary Tables 3C, D** for complete DE results. ^2^L2FC, log2fold change. FDR^3^ = Adjusted p-values with Benjamini-Hochberg correction for false discovery rate. ^4^ENSBTAG and LOC notations are used for novel uncharacterized genes only.

**Figure 2:**
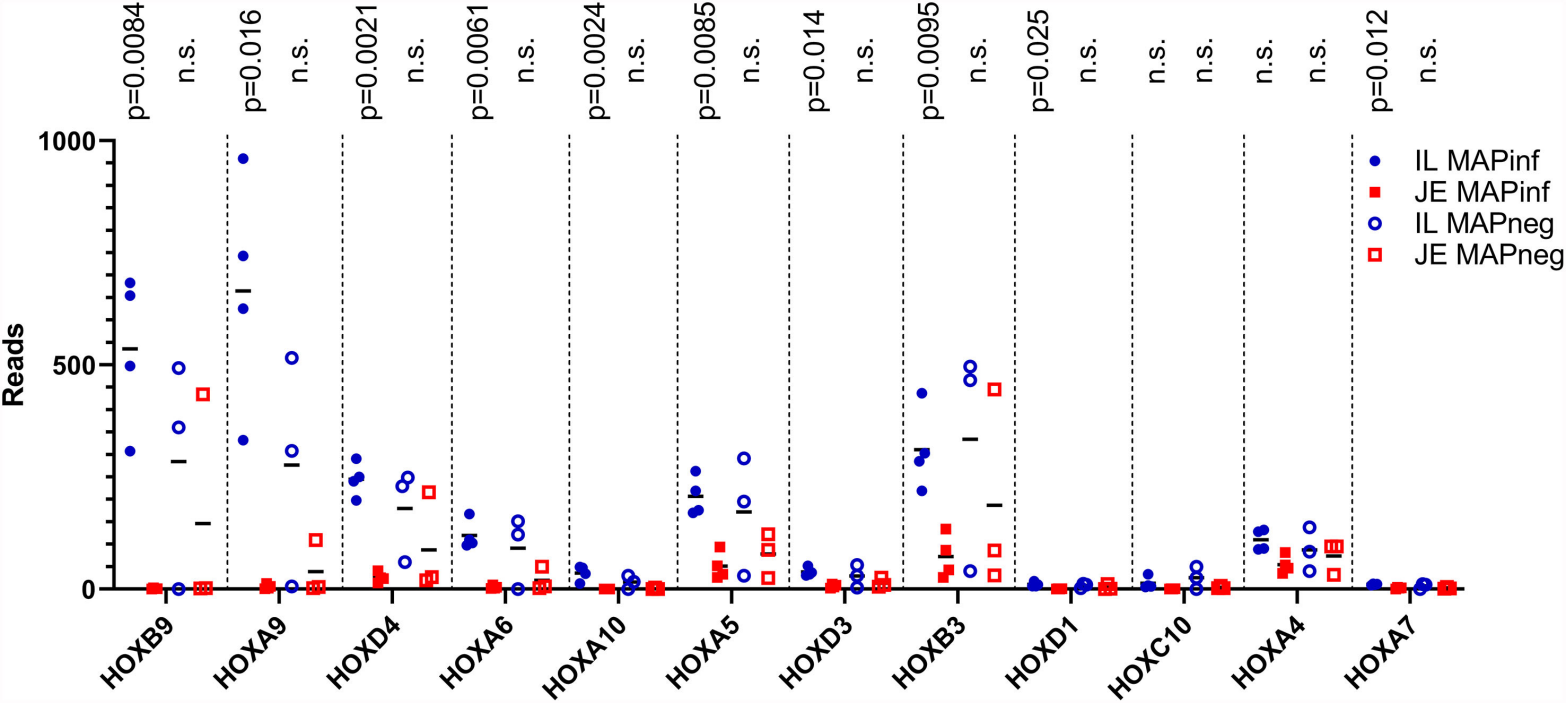
HOX genes expression in IL and JE of MAP-infected cows compared with IL and JE MAP-negative cows. ns, not significant.

To identify potential differences between the IL and JE MAPinf tissues and between MAPneg IL and JE tissues, DE analysis was performed. Comparison of the IL MAPinf and JE MAPinf identified a total of 65 significant DE genes (**Supplementary Table S3E**). Among the 65 DE genes, genes of the HOX family including *HOXB9*, *HOXA9*, *HOXA6, HOXD4, HOXA10, HOXA5* and *HOXB3* were among the top DE genes (**Table 4** and **Figure 2**). No significant DE gene was found between IL MAPneg and JE MAPneg or between their associated lymph nodes (ILLN MAPneg and JELN MAPneg) or between lymph node from MAPinf cows (ILLN MAPinf and JELN MAPinf).

**Table 4:** Top differentially expressed genes, including genes of the HOX family, between ileum MAP-infected and jejunum MAP-infected tissues^1^.

Gene	Base mean	L2FC^2^	P-value	FDR^3^	Function
**HOXB9**	268.6139	8.72	3.50E-32	5.96E-28	Myeloid transcriptional regulator.
ENSBTAG00000011476 (**HOXA9**)	335.4423	6.93	2.06E-28	1.75E-24	Play a role in the innate immune response to bacterial infection as a modulator of NF-κB-dependent transcription.
**HOXD4**	135.4938	3.24	6.30E-24	3.58E-20	Play a role in determining positional values in developing limb buds.
NPC1L1	653.5329	-5.62	3.40E-23	1.45E-19	Role in intestinal epithelial cells and cholesterol transport.
**HOXA6**	61.99351	4.79	6.42E-21	2.19E-17	Myeloid transcriptional regulator.
TSPAN1	1090.086	5.11	1.39E-18	3.94E-15	Roles in cell development, activation, growth and motility.
ASS1	1834.547	3.60	2.92E-16	7.11E-13	Role in urea cycle function in processing excess nitrogen that is generated as the body breaks down proteins.
Bta-mir-196a-1	45.61055	8.22	3.18E-14	6.77E-11	Roles in disease.
**HOXA10**	18.02714	7.60	2.50E-10	4.74E-07	Regulator of normal and malignant hematopoiesis.
ST8SIA6	122.4066	6.30	3.14E-10	4.87E-07	Supports alpha-N-acetylneuraminate alpha-2,8-sialyltransferase activity; sialyltransferase activity.
APOA4	778.1787	-3.77	2.94E-10	4.87E-07	Roles in antioxidant activity, cholesterol binding, cholesterol transfer and phosphatidylcholine binding, etc.
PITX2	41.00817	-2.88	9.12E-09	1.23E-05	Supports DNA-binding transcription factor activity, RNA polymerase II-specificity; sequence-specific double-stranded DNA binding and transcription cis-regulatory region binding.
ENSBTAG00000017233	1269.108	1.82	9.39E-09	1.23E-05	Novel gene.
GATA4	88.92491	-3.25	2.24E-08	2.73E-05	Roles in DNA binding; DNA-binding transcription activator activity and NFAT protein binding,etc.
bta-mir-10b	19.42987	2.60	2.48E-08	2.82E-05	No information available.
MME	1415.124	-3.24	3.58E-08	3.39E-05	Roles in cardiolipin binding; endopeptidase activity; exopeptidase activity and metalloendopeptidase activity, etc.
NPNT	852.933	1.73	3.43E-08	3.39E-05	Roles in calcium ion binding; extracellular matrix structural constituent and integrin binding.
SLC5A8	470.0292	4.25	3.44E-08	3.39E-05	Roles in lactate transmembrane transporter activity; monocarboxylate:sodium symporter activity; organic acid:sodium symporter activity; propionate transmembrane transporter activity and symporter activity.
**HOXA5**	129.4717	2.01	1.84E-06	0.001163	May play an important role in tumorigenesis.
ENSBTAG00000027225 [LAP (lingual antimicrobial peptide)]	90.92925	6.42	2.40E-06	0.001462	Shows a broad spectrum of antibacterial and antifungal activities.
**HOXD3**	22.86329	2.48	2.69E-06	0.001581	May play a role in the regulation of cell adhesion processes.
**HOXB3**	192.2567	2.10	3.03E-05	0.011715	A transcription factor involved in development.

^1^See **Supplementary Table 3E** for complete DE results. L2FC^2^, log2fold change. FDR^3^ , Adjusted p-values with Benjamini-Hochberg correction for false discovery rate.

### Biological Events, Pathways, and Gene Networks in Ileum and Jejunum of Subclinical JD Cows

Knowledge of the possible biological activities associated with the DE genes were gained through GO and pathways analyses. Analysis of the 585 DE genes in IL of MAPinf identified 658 biological processes (BP), 39 cellular component (CC), 62 molecular function (MF) GO terms, and 44 KEGG pathways as significantly enriched (FDR<0.05) (**Supplementary Tables S4A, B**). In JE of MAPinf, the 218 DE genes were enriched in 69 BP GO terms, and 6 MF GO terms while no CC GO term was enriched (**Supplementary Table S5A**). A total of 46 BP GO terms were common to the IL and JE while 612 and 23 BP GO terms were unique to the IL and JE, respectively (**Supplementary Tables S4C** and **Figure 1B**). The 20 top enriched BP GO terms in the IL and JE are listed in **Table 5**. 64% (n=420) of enriched BP GO terms in the IL are related to immune processes and disease (**Supplementary Table S4A**) and 90% of the immune/disease BP GO terms were enriched for with up-regulated genes (**Supplementary Tables S3A** and **S4A**). By contrast, only 41 (59%) of JE enriched BP GO terms were of the immune and disease classes (**Supplementary Table S5A**). Similarly, more IL BP GO terms (19%) were enriched for metabolic processes as compared to 10% BP GO terms for JE (**Supplementary Tables S4A** and **S5A**). Interestingly, three BP GO terms (myeloid leukocyte migration, leukocyte chemotaxis and monocyte chemotaxis) were common to all tissues analysed (IL, JE, ILLN and JELN) while 46 BP GO terms were common to IL and JE tissues (**Supplementary Tables S4C, D**). IL enriched MF GO terms were mostly involved in binding activities (**Supplementary Table S4A**). The top three IL CC GO terms were cell surface (FDR=1.22E-11), external side of plasma membrane (FDR=1.27E-11) and side of membrane (FDR=3.47E-10) (**Supplementary Table S4A**). The most enriched JE MF GO terms included low-density lipoprotein particle binding (FDR=0.0002), Hsp90 protein binding (FDR=0.0096) and fatty acid binding (FDR=0.0061) (**Supplementary Table S5A**).

**Table 5:** Twenty top enriched biological processes gene ontology (GO) terms for ileum and jejunum differentially expressed genes^1^.

ID	Biological process gene ontology term	P-value	FDR^2^	% Associated Genes	# Genes
**Ileum**					
GO:0006955	Immune response	0	0	12.14	153
GO:0006952	Defense response	6.726E-44	2.93E-41	12.06	130
GO:0009607	Response to biotic stimulus	9.654E-36	2.81E-33	11.47	113
GO:0051707	Response to other organism	5.99E-35	1.31E-32	11.53	110
GO:0002682	Regulation of immune system process	2.473E-33	4.31E-31	10.81	113
GO:0009605	Response to external stimulus	2.336E-30	3.39E-28	8.06	151
GO:0098542	Defense response to other organism	4.901E-30	6.11E-28	12.24	89
GO:0045087	Innate immune response	4.948E-27	5.39E-25	13.94	70
GO:0050776	Regulation of immune response	1.397E-26	1.35E-24	12.67	76
GO:0006954	Inflammatory response	5.748E-26	5.01E-24	13.99	67
GO:0045321	Leukocyte activation	5.913E-25	4.69E-23	11.68	78
GO:0001775	Cell activation	4.849E-24	3.52E-22	10.83	82
GO:0034097	Response to cytokine	2.034E-21	1.36E-19	10.83	73
GO:0002684	Positive regulation of immune system process	3.501E-21	2.18E-19	11.11	70
GO:0046649	Lymphocyte activation	3.915E-21	2.28E-19	11.66	66
GO:0070887	Cellular response to chemical stimulus	6.351E-21	3.46E-19	6.60	149
GO:0006950	Response to stress	1.068E-19	5.48E-18	6.10	164
GO:0071345	Cellular response to cytokine stimulus	5.171E-19	2.5E-17	10.65	66
GO:0009617	Response to bacterium	1.886E-18	8.65E-17	12.50	53
GO:0034341	Response to interferon-gamma	5.598E-17	2.44E-15	25.00	26
**Jejunum**					
GO:0009617	Response to bacterium	1.03E-10	7.21E-09	5.66	24
GO:0006954	Inflammatory response	1.21E-09	4.24E-08	5.01	24
GO:0042742	Defense response to bacterium	6.64E-07	1.55E-05	6.34	13
GO:0060326	Cell chemotaxis	1.45E-06	2.54E-05	5.47	14
GO:0038024	Cargo receptor activity	2.16E-06	3.02E-05	10.67	8
GO:0006959	Humoral immune response	6.28E-06	6.28E-05	6.18	11
GO:0030595	Leukocyte chemotaxis	5.64E-06	6.58E-05	6.25	11
GO:0097529	Myeloid leukocyte migration	1.69E-05	0.000148	6.17	10
GO:0005044	Scavenger receptor activity	2.38E-05	0.000151	11.76	6
GO:0050900	Leukocyte migration	2.18E-05	0.000152	4.61	13
GO:0019730	Antimicrobial humoral response	2.11E-05	0.000164	9.46	7
GO:0002532	Production of molecular mediator involved in inflammatory response	3.68E-05	0.000172	10.91	6
GO:0007159	Leukocyte cell-cell adhesion	3.02E-05	0.000176	4.47	13
GO:0032496	Response to lipopolysaccharide	3.61E-05	0.000181	5.65	10
GO:1904417	Positive regulation of xenophagy	4.92E-05	0.000215	42.86	3
GO:0071216	Cellular response to biotic stimulus	5.31E-05	0.000219	6.04	9
GO:0098869	Cellular oxidant detoxification	6.99E-05	0.000272	7.87	7
GO:0071222	Cellular response to lipopolysaccharide	8.27E-05	0.000305	6.50	8
GO:0032757	Positive regulation of interleukin-8 production	0.000138	0.000483	11.36	5
GO:0061844	Antimicrobial humoral immune response mediated by antimicrobial peptide	0.000154	0.000512	11.11	5

^1^See **Supplementary Tables S4A** and **S5A** for complete list of significantly enriched GO terms. FDR^2^ = Adjusted p-values with Benjamini-Hochberg correction for false discovery rate.

**Figure 3:**
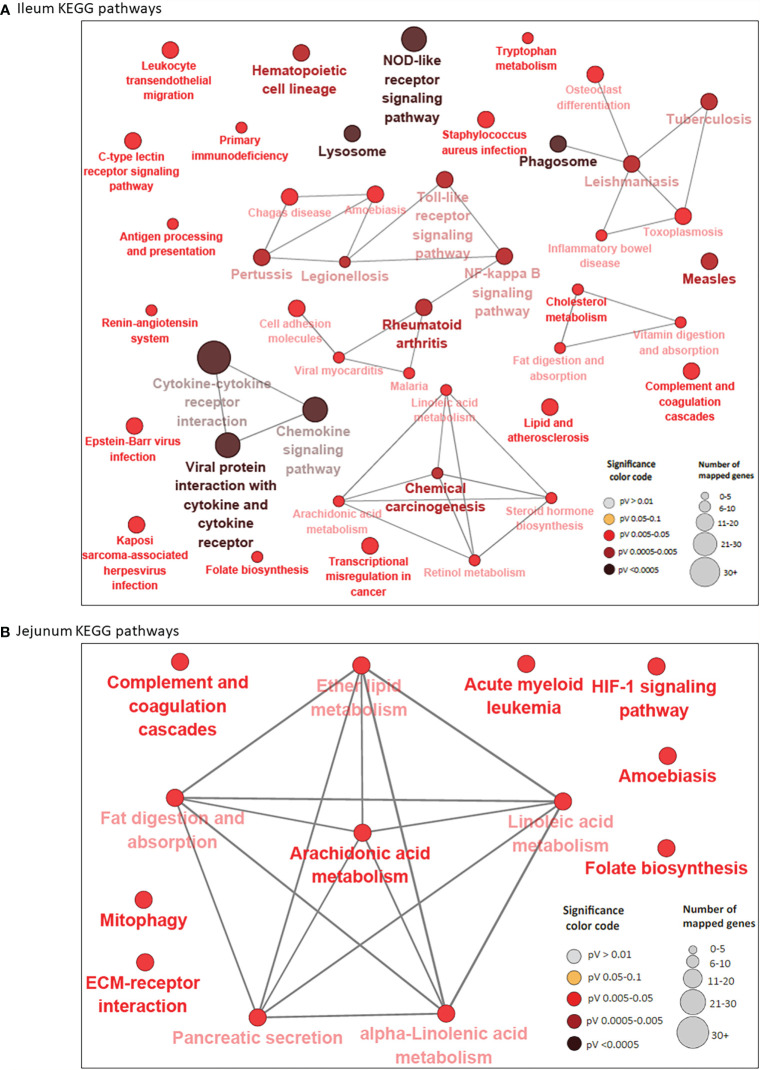
KEGG Pathways enriched for differentially expressed genes in ileum **(A)** and jejunum **(B)** showing interactions between pathways. Each node represents a pathway and the color of the node represents the level of significance (the deeper the color of a node, the higher the level of significance). The size of the node represents the number of mapped genes (the bigger the node, the higher the number of mapped genes). Straight lines connects pathways.

A total of 44 and 13 KEGG pathways were enriched among the IL and JE DE genes, respectively (**Supplementary Tables S4B** and **S5B**). Interactions between IL and JE enriched KEGG pathways are shown in **Figure 3**. Interestingly, 77% of IL pathways were enriched with up-regulated genes, meanwhile, only three of the 13 JE KEGG pathways were of the disease/immune class including two (Amoebiasis and Acute myeloid leukemia) enriched with up-regulated genes. The most enriched pathways in the IL included Viral protein interaction with cytokine and cytokine receptor (FDR=3.11E-10), Chemokine signaling pathway (FDR=2.73E-07), Cytokine-cytokine receptor interaction (FDR=2.97E-07), NOD-like receptor signalling pathway (FDR=1.37E-06), Rheumatoid arthritis (FDR=5.47E-04), Lysosome (FDR=1.72E-04), and Phagosome (FDR = 3.57E-04). Other notable enriched pathways in the IL included NF-κB signalling, Toll-like receptor signaling pathway, Leukocyte transendothelial migration, Leishmaniasis and Tuberculosis signalling pathways (**Supplementary Table S4B**). Out of the IL pathways mostly enriched with up-regulated genes, 97% were immune and disease related pathways with notable examples as Chemokine signaling pathway, NF-κB signaling pathway, Toll-like receptor signaling pathway, Leukocyte transendothelial migration, Leishmaniasis, Phagosome, Amoebiasis, Tuberculosis, Legionellosis, Inflammatory bowel disease and C-type lectin receptor signaling pathway, among others. Meanwhile, 8 out of 9 metabolic/biosynthetic IL pathways (Folate biosynthesis, Fat digestion and absorption, Vitamin digestion and absorption, Linoleic acid metabolism, Retinol metabolism, Arachidonic acid metabolism, Tryptophan metabolism and Steroid hormone biosynthesis) were enriched for with down-regulated genes (**Supplementary Table S4B**). Notables of the seven JE pathways with functions in metabolism/biosynthesis were Fat digestion and absorption, Arachidonic acid metabolism (enriched with down-regulated genes), Alpha-linolenic acid metabolism and Ether lipid metabolism (**Supplementary Table S5B**). The most enriched JE KEGG pathways were Arachidonic acid metabolism (FDR=0.0067), Acute myeloid leukemia (FDR=0.0076) and HIF-1 signaling (FDR=0.0076) pathways.

While 38 pathways were exclusive to IL and seven to JE, six were common to the two tissues including two immune/disease pathways (Amoebiasis and Complement and coagulation cascades) and four metabolic pathways (Folate biosynthesis, Linoleic acid metabolism, Arachidonic acid metabolism and Fat digestion and absorption (**Supplementary Table S4E** and **Figure 1C**).

A total of 52 genes were considered pathway driving genes as they were enriched in four or more IL pathways including *TLR4* (16 pathways), *CXCL8* (15 pathways), *NF-κBIA* (14 pathways), *IL12B* (13 pathways), *ITGB2* (13 pathways), *BOLA-DOA* (12 pathways), *STAT1* (12 pathways), *GRO1* (10 pathways), *ICAM1* (10 pathways) and *CD14* (10 pathways) (**Supplementary Table S4F**). On the other hand, only three pathway driving genes (*LOC615045, PLA2G2A* and *PLA2G5* (each enriched in six pathways) were identified in the JE (**Supplementary Table S5C**).

**Figure 4:**
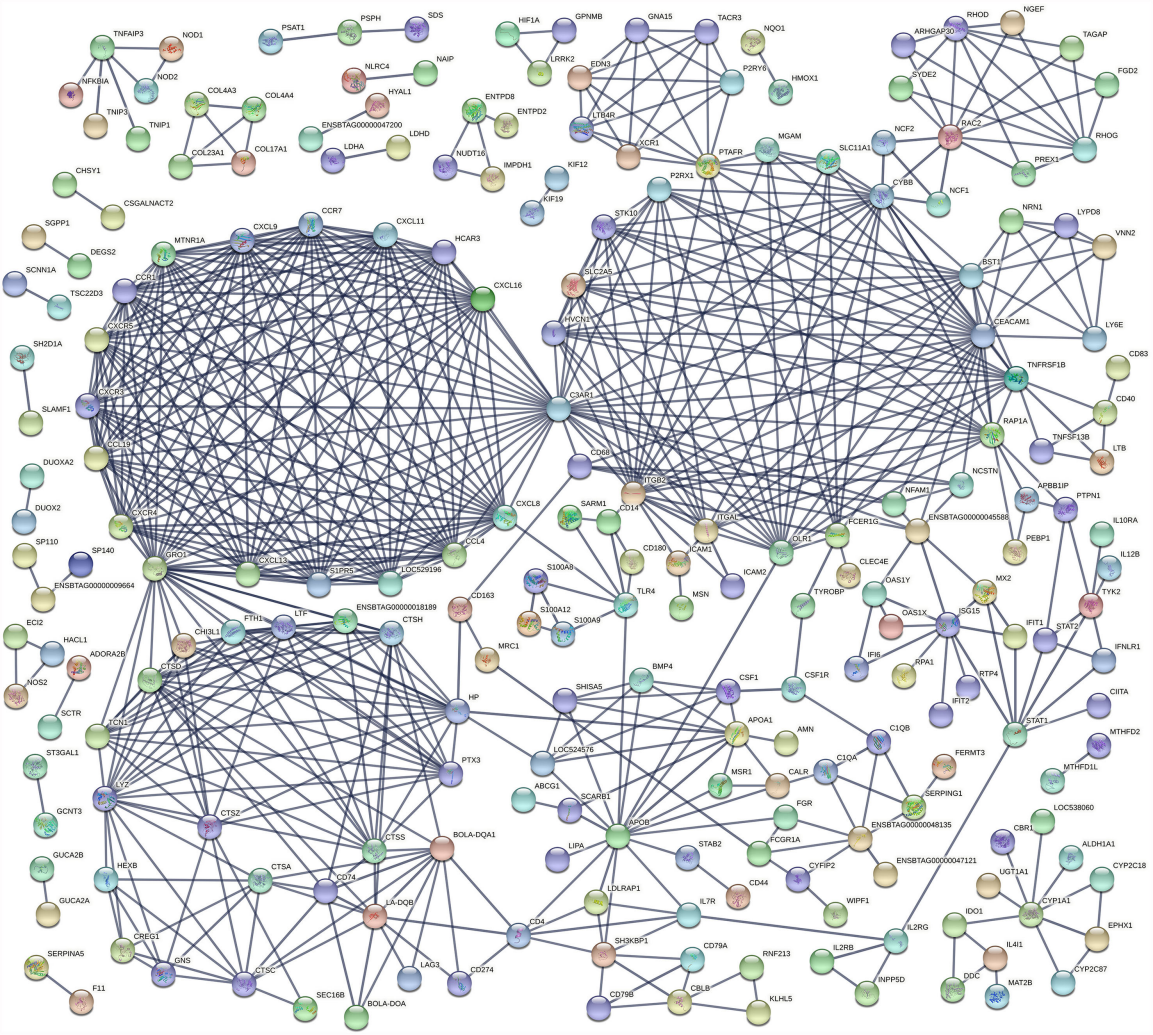
Gene interaction networks between differentially expressed genes in the ileum.

An analysis of the interaction between DE genes (PPI network) indicated that 225 DE genes in the IL interacted with at least one or more other DE genes (**Figure 4**) while 65 JE DE genes interacted with at least one other gene (**Supplementary Figure S1**). In particular, three major networks of interactions among genes of the chemokine receptors, inflammation/apoptosis/integrin genes and MHC-antigens/immune response genes were identified in the IL. The main hub genes or genes that interacted the most with other IL DE genes included *C3AR1, GRO1, CEACAM1, CYBB, ITGB2, CXCL8, APOB, CCL19, CCL4, CCR1, CCR7, CXCL11, CXCL13* and *CXCL16*. In the JE, three main gene-gene interaction networks were identified. The genes with the most interactions or hub genes in the JE were *ENSBTAG00000006859, CD33, PTAFR, MCEMP1* and *OLR1*.

### Biological Events, Pathways, and Gene Networks in Ileal and Jejunal Lymph Nodes From Subclinical JD Cows

The 117 ILLN (MAPinf) DE genes were significantly enriched in 10 BP, one MF and zero CC GO terms (**Supplementary Table S6A**). The majority of enriched (FDR<0.05) BP GO terms were related to B cell functions and movement of cells. These included regulation of B cell activation, positive regulation of B cell activation, regulation of lymphocyte proliferation, leukocyte chemotaxis, granulocyte chemotaxis, monocyte chemotaxis and lymphocyte migration, etc. (**Supplementary Table S6A**). The top 20 enriched BP GO terms in the ILLN and JELN are shown in **Table 6**.

**Table 6:** Biological processes gene ontology (GO) terms enriched for ileum lymph node differentially expressed (DE) genes and top 20 biological processes GO terms enriched for jejunum lymph node DE genes^1^.

GOID	Biological process GO term	P-value	FDR^2^	% Associated Genes	# Genes
**Ileum lymph node**					
GO:0030595	Leukocyte chemotaxis	1.02E-05	5.63E-05	4.55	8
GO:0071621	Granulocyte chemotaxis	2.45E-05	9E-05	6.19	6
GO:0050670	Regulation of lymphocyte proliferation	8.65E-06	9.51E-05	4.65	8
GO:0050864	Regulation of B cell activation	3.64E-05	0.0001	5.77	6
GO:0062099	Negative regulation of programmed necrotic cell death	5.62E-05	0.000103	23.08	3
GO:0097529	Myeloid leukocyte migration	5.18E-05	0.000114	4.32	7
GO:0002548	Monocyte chemotaxis	0.00016	0.000251	8.70	4
GO:0050871	Positive regulation of B cell activation	0.000721	0.000881	5.88	4
GO:0097300	Programmed necrotic cell death	0.001375	0.001513	8.11	3
GO:0072676	Lymphocyte migration	0.002218	0.002218	4.35	4
**Jejunum lymph node**					
GO:0042554	Superoxide anion generation	7.46E-06	8.58E-05	19.23	5
GO:0032941	Secretion by tissue	1.1E-05	0.000101	17.86	5
GO:0019318	Hexose metabolic process	0.000134	0.000617	5.06	9
GO:0031663	Lipopolysaccharide-mediated signaling pathway	0.000117	0.000675	11.11	5
GO:0070254	Mucus secretion	0.000189	0.000726	27.27	3
GO:0070482	Response to oxygen levels	0.000403	0.001324	4.88	8
GO:0030595	Leukocyte chemotaxis	0.000643	0.001973	4.55	8
GO:0007589	Body fluid secretion	0.000821	0.00236	7.35	5
GO:0002237	Response to molecule of bacterial origin	0.00117	0.002833	4.15	8
GO:0016052	Carbohydrate catabolic process	0.001155	0.002952	5.50	6
GO:0001666	Response to hypoxia	0.00111	0.003004	4.73	7
GO:0030593	Neutrophil chemotaxis	0.001525	0.00334	6.41	5
GO:0097529	Myeloid leukocyte migration	0.001867	0.003735	4.32	7
GO:0072593	Reactive oxygen species metabolic process	0.002072	0.003972	4.24	7
GO:0002221	Pattern recognition receptor signaling pathway	0.002828	0.005204	4.62	6
GO:0071219	Cellular response to molecule of bacterial origin	0.002828	0.005204	4.62	6
GO:0006006	Glucose metabolic process	0.004519	0.007423	4.20	6
GO:0071674	Mononuclear cell migration	0.004219	0.007465	4.26	6
GO:0006778	Porphyrin-containing compound metabolic process	0.004406	0.007507	9.68	3
GO:0016051	Carbohydrate biosynthetic process	0.004833	0.007666	4.14	6

^1^See **Supplementary Tables S6A** and **S7A** for complete list of significantly enriched GO terms. FDR^2^ = Adjusted p-values with Benjamini-Hochberg correction for false discovery rate.

The higher number of DE genes found in the JELN of MAPinf cows translated to a higher number of enriched BP (34), CC (6) and MF (8) GO terms when compared with the ILLN (**Supplementary Table S7A**). About 36% of the JELN BP GO terms are related to various immune processes including lipopolysaccharide-mediated signalling pathway, leukocyte chemotaxis, response to molecule of bacterial origin, neutrophil chemotaxis, pattern recognition receptor signalling pathway, defence response to fungus, regulation of mononuclear cell migration and lymphocyte migration, etc. The most enriched JELN BP GO terms were superoxide anion generation (FDR=8.58E-05), secretion by tissue (FDR=1.07E-04), hexose metabolic process (FDR=6.17E-04) and lipopolysaccharide-mediated signalling pathway (FDR=6.75E-04) (**Tables 6** and **Supplementary Table S7A**). Surprisingly, only four BP GO terms (leukocyte chemotaxis, myeloid leukocyte migration, monocyte chemotaxis and lymphocyte migration) were common to the ILLN and JELN tissues (**Supplementary Table S6C** and **Figure 1B**). While no enriched BP GO term was related to the metabolic process in the ILLN, about 8 (24%) BP GO terms in the JELN were related to various metabolic processes including hexose metabolic process, carbohydrate catabolic process, glucose metabolic process, reactive oxygen species metabolic process, carbohydrate biosynthetic process and hexose biosynthetic process. The only enriched MF GO term in the ILLN was chemokine receptor binding (FDR=6.15E-04) while the most enriched CC and MF GO terms in the JELN were NADPH oxidase complex (FDR=4.67E-06) and superoxide-generating NAD(P)H oxidase activity (FDR=1.24E-05), respectively (**Supplementary Tables S6A** and **S7A**).

**Figure 5:**
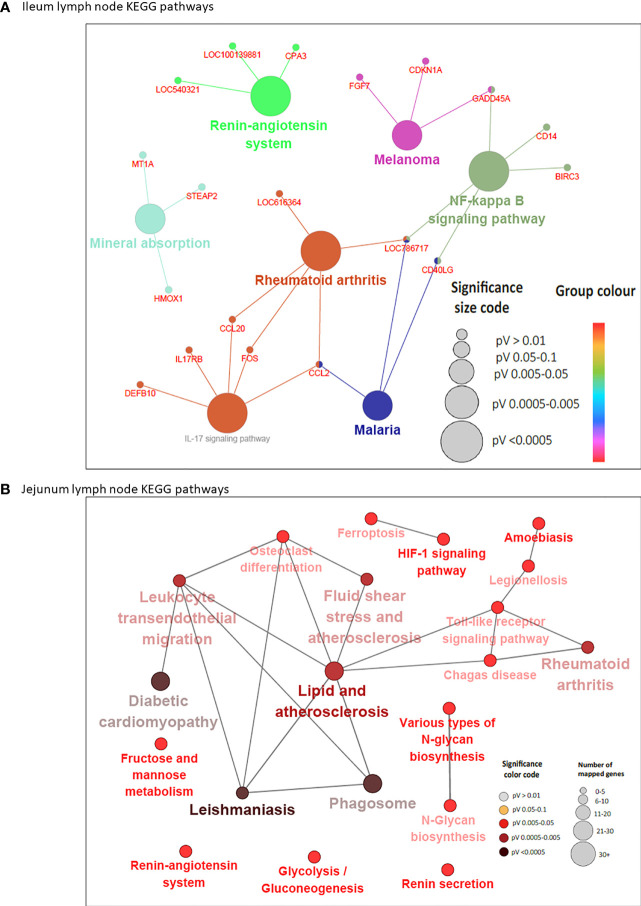
KEGG Pathways enriched for differentially expressed genes in ileal lymph node **(A)** and jejunum lymph node **(B)** showing interactions between genes shared by pathways and connections between pathways. Each node (large) represents a pathway and straight lines connects genes and pathways. In **(A)**, the size of the node represents the level of significance (the larger the node, the higher the level of significance). Small nodes represent individual genes. In **(B)**, the color and size of the node represents the level of significance and number of mapped genes, respectively.

In concordance with the number of DE genes identified, more KEGG pathways (20) were enriched among the 205 JELN DE genes than among the 117 ILLN DE genes (7 KEGG pathways) (**Supplementary Tables S6B** and **S7B**). Interactions among the ILLN and JELN KEGG pathways are shown in **Figure 5**. Three (IL-17 signaling pathway, Malaria and Renin-angiotensin system) out of seven ILLN KEGG pathways were enriched with down-regulated genes while the other four (NF-*κ*B signaling pathway, Mineral absorption, Melanoma and Rheumatoid arthritis) were enriched with both up- and down-regulated genes (**Supplementary Table S6B**). While only five of the seven ILLN enriched pathways are associated with disease conditions and the immune response (**Figure 5**), 11 (55%) JELN KEGG pathways have immune/disease related functions, including six (Leishmaniasis, Diabetic cardiomyopathy, Phagosome, Leukocyte transendothelial migration, Ferroptosis and Legionellosis) enriched for with up-regulated genes. Interestingly, four metabolic processes related pathways were enriched for JELN DE genes (Glycolysis/Gluconeogenesis, Fructose and mannose metabolism, Various types of N-glycan biosynthesis and N-Glycan biosynthesis) while none was enriched for ILLN DE genes (**Supplementary Tables S6B** and **S7B**). Pathways involved in the regulation of oxygen homeostasis were also enriched in this study including HIF-1 signaling pathway (JELN). Only two pathways were common to the ILLN and JELN (Renin-angiotensin system and Rheumatoid arthritis pathways) (**Supplementary Table S6D** and **Figure 1C**).

Six DE genes (*CCL20, CD40LG, FOS, GADD45A, LOC786717 and CCL2*) where shared by two or three pathways in the ILLN while 10 DE genes in the JELN were common to four or more pathways including *TLR2* (eight pathways), *NCF1* (seven pathways), *NCF2* (seven pathways), *NCF4* (six pathways), *CYBA* (seven pathways), *CYBB* (seven pathways) and *FOS* (seven pathways) (**Supplementary Tables S6E** and **S7C**). Gene-gene interaction network (PPI network) for ILLN DE genes showed that 25 DE genes interacted with at least one other gene (**Supplementary Figure S2A**) including two main hub genes, *GNG7* and *IGLL1*, which interacted with five (*CCL20, CYSLTR2, HRH1, ADCYAP1R1*, *GPR15*) and three (*MZB1, ANSBTAG00000003408, ANSBTAG000000047529*) other genes, respectively. In the JELN, 53 DE genes interacted with at least one other gene (**Supplementary Figure S2B**) including *VAMP8*, *CYBB*, *CYBA*, *DDOST* and *P4HB* which interacted with 10 to 20 other genes other genes, and were considered the main hub genes.

### Immunohistochemical Staining for Myeloid, T Cells Markers, and Proliferating Cells

**Figure 6:**
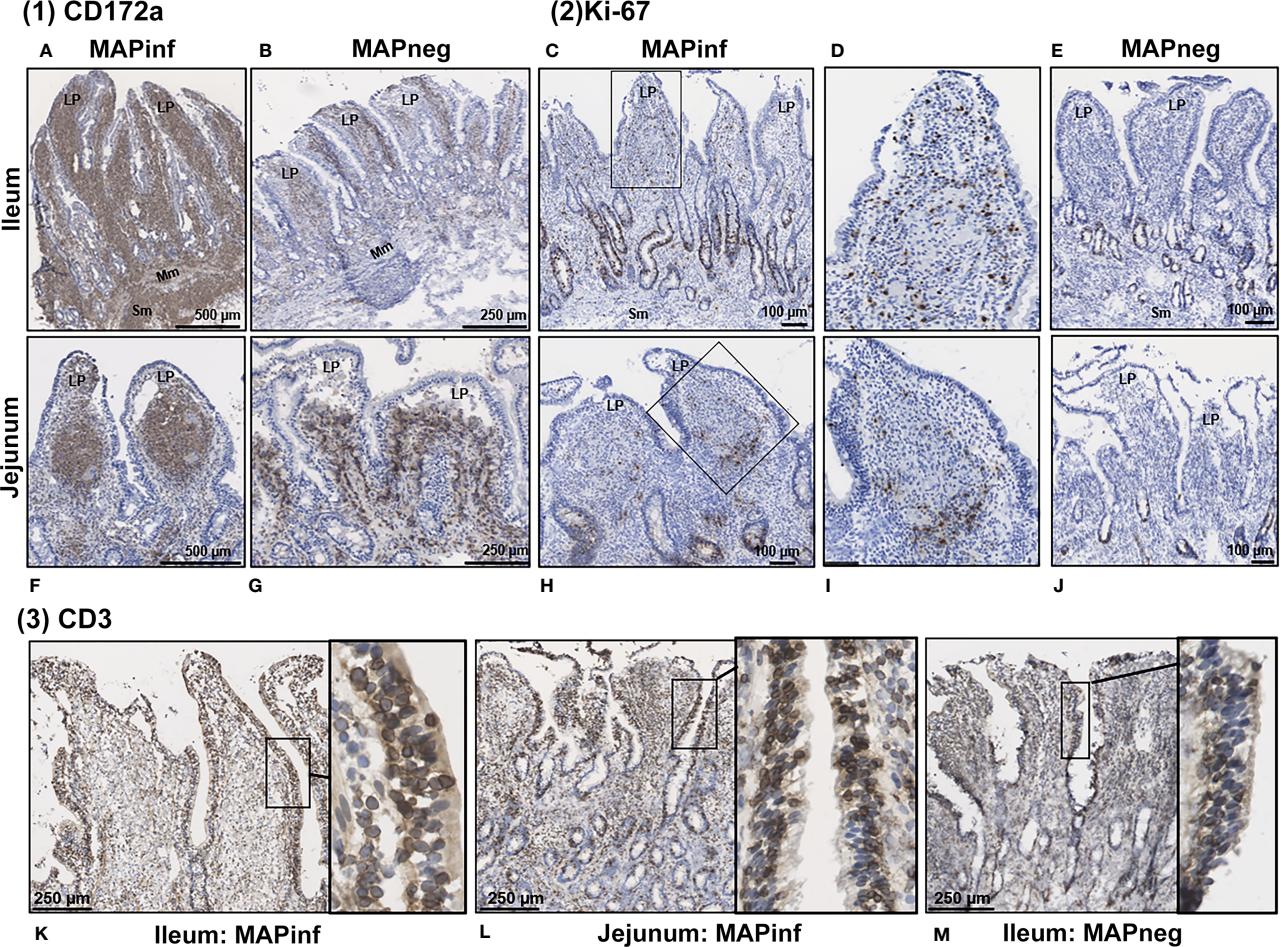
Immunohistochemical staining of intestinal tissues from MAP-infected and uninfected cows. (1) Representative tissue sections stained for CD172a myeloid cells (brown stain) from MAP-infected **(A, F)** and uninfected **(B, G)** ileum **(A, B)** and jejunum **(F, G)**. (2) Staining for Ki-67 (PCNA) in intestinal tissue (brown stain) from MAP-infected **(C, H)** and uninfected **(E, J)** ileum **(C‒E)** and jejunum **(H‒J)**. Panels **(D**, **I)** are digital magnification of boxed regions in panels **(C, H)**, respectively. (3) Representative ileum and jejunum tissue sections from MAP-infected **(K, L)** and uninfected **(M)** cow stained for the pan T-cell surface marker CD3 (brown stain). Insets in each panel are digital magnifications of boxed areas showing the circumferential staining of CD3 on intraepithelial cells in MAP-infected **(K, L)** tissue sections compared to a non-infected **(M)** tissue. LP, lamina propria; Mm, muscularis mucosa; SM, submucosa.

Transcriptional profiling of intestinal tissues revealed an increased abundance of *CD14* reads in both IL and JE MAPinf tissues when compared to their respective controls (**Supplementary Figure S3**). To further validate this, we performed immunohistochemical staining of tissue sections with CD172a-specific antibodies, which stains macrophages and other myeloid cells, including dendritic cells, in the bovine intestine (21). In IL of MAPinf cows, CD172a staining was abundant and diffuse throughout the LP and submucosa (**Figure 6A**). In contrast, CD172a staining in JE of MAP-infected cows was organized into focal aggregates in the LP, suggestive of granulomatous-like lesions but staining was diffuse in the submucosa (**Figure 6F**). CD172a staining was visually much less abundant in both these intestinal compartments within uninfected tissues (**Figure 6B, G**). Transcriptional analyses also suggested an increased abundance of homeobox (*HOX)* genes in IL when compared to JE from MAPinf cows (**Figure 2** and **Supplementary Table S3E**). The up-regulation of these genes, specifically *HOXB9* and *HOXA9* homologs in other species, have been associated with cell transformation in myeloid leukemia and altered functional states in lymphocytes. To further investigate whether cells in the LP were in an activated state, tissue sections were stained for Ki-67 (i.e. nuclear antigen expressed in activated or proliferating cells). In MAPinf IL tissue, Ki-67 staining was dispersed throughout the LP (**Figures 6C, D**). By contrast, in MAPinf JE tissue, Ki-67 staining was also present in the LP but localized primarily to the periphery of regions that had stained intensely with CD172a (**Figures 6H, I**). In uninfected tissues, Ki-67 staining localized exclusively to crypt epithelium and was absent in the LP (**Figures 6E, J**). These observations are consistent with the proliferative state of intestinal crypt epithelial cells, and the presence of terminally-differentiated effector cells throughout the LP. Moreover, in Ki-67 stained tissues, cytoplasmic-dense regions with visually less nuclei were observed in the LP of MAPinf IL and JE (compare **Figures 6D, I** to **Figures 6E, J**); these regions had stained abundantly with CD172a.Thus, in IL and JE LP of MAP-infected cows, Ki-67 staining revealed activated cells with a distinct staining pattern in each tissue. In JE LP, Ki-67 stained cells localized at the margins of areas staining intensely for CD172a, but in IL Ki-67 stained cells were distributed throughout the LP where CD172a cells were also abundant.

Staining for T cells (CD3) was observed in the LP and within the mucosal epithelial layer of MAPneg animals (**Figure 6M**) while no visible staining for CD11c was observed in all the studied tissues (**Supplementary Figure S4**). CD3 staining is consistent with approximately 60% of the cell population in the LP being T-cells (**Figure 6M**) (34). Based on observations with intestinal tissue sections from two MAPinf cows, there was increased staining density for CD3 within the epithelium when compared to MAPneg cows (**Figures 6K, L**).

**Figure 7:**
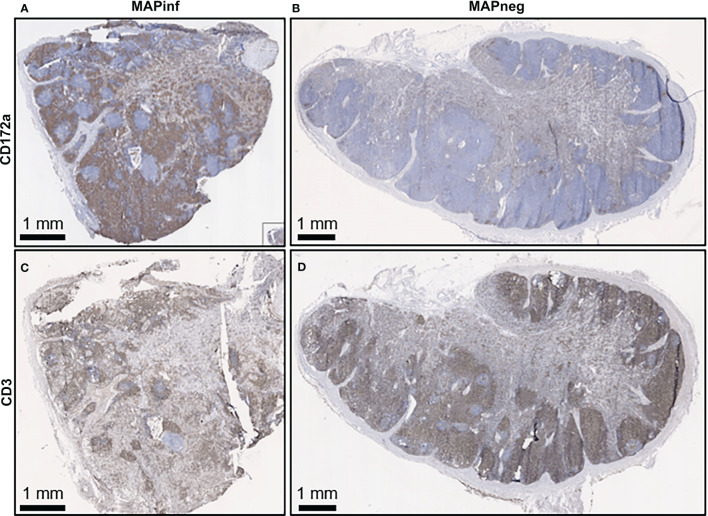
Immunohistochemical staining for CD172a myeloid cells and CD3 T lymphocytes in ileal mesenteric lymph nodes. Representative mesenteric lymph node tissue sections from MAP-infected cows **(A, C)** and MAP negative cows **(B, D)** stained for the myeloid cell-surface marker CD172a (brown stain) **(A, B)** and pan T-cell surface marker CD3 (brown stain) **(C, D)**.

IHC staining of mesenteric lymph nodes (ILLN and JELN) indicated that CD172a cells appeared as discrete foci throughout the normal lymph node (MAPneg cow) (**Figure 7B**), while as expected, CD3 staining was abundant throughout the lymph node cortex (**Figure 7D**). Lymph nodes from MAPinf cows displayed a substantial increase in CD172a staining density when compared to MAPneg animals (**Figures 7A, B**). Increased focal staining for myeloid cells in mesenteric lymph node of cows with JD is not unexpected (35) but the extensive myeloid cell infiltration that we observe is surprising. This extensive staining may be a consequence of using the CD172a mAb that stains not only macrophages but other myeloid cells, such as dendritic cells, in the bovine intestine (21). The pattern of CD172a staining and examination of H&E stained sections reveals that this myeloid cell infiltration is diffuse throughout the cortex and medulla of the lymph node and macrophages are not aggregated in granulomas. This extensive lymph node infiltration by myeloid cells may therefore represent increased myeloid cells trafficking from the LP to the draining lymph node through the afferent lymphatics (36, 37). Without further characterization of the specific myeloid cell populations present in the mesenteric lymph node it is difficult to speculate what the functional consequences of this myeloid cell accumulation may be. RNA-Seq data confirms significant alterations in mesenteric lymph node transcriptome but single cell transcriptome analysis would be required to directly link specific changes with myeloid cells. A decrease in CD3 staining was also observed in lymph node sections from MAPinf cows when compared to MAPneg samples (**Figures 7C, D**).

### Quantitative Real Time PCR Validation for Selected Genes

**Figure 8:**
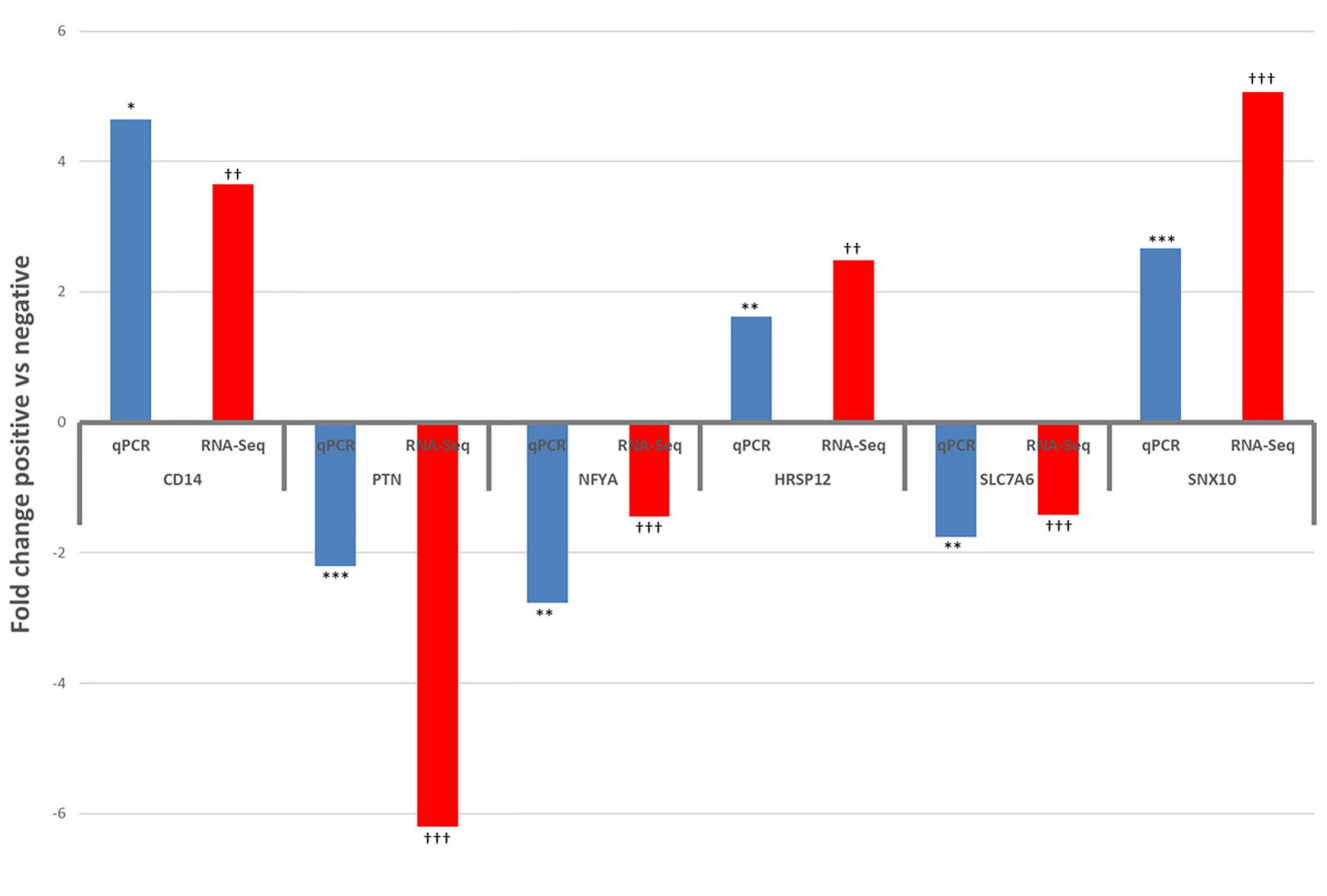
Results of qPCR validation of the expression of differentially expressed genes in the ileum lymph node (CD14 and PTN) and jejunum lymph node (NFYA, HRSP12, SLC7A6 and SNX10), compared with RNA-Seq results. qPCR: *P<0.05, **P<0.01, ***P<0.001. RNA-Seq: ^†^P<0.05, ^††^P<0.01, ^†††^P<0.001.

Six DE and four non-DE genes were randomly selected from ILLN and JELN DE genes for verification by qPCR. Two (ILLN) and four (JELN) DE genes as well as 4 non-DE genes (ILLN and JELN) were tested. Out of the four non-DE genes tested, the most stably expressed genes in both tissues (*ATP5B* and *RPS12*) were used as housekeeping genes. Following normalization, all genes that were not DE based on RNA-Seq results were also not DE by qRT-PCR (data not shown). However, all genes identified as DE by RNA-Seq where confirmed as DE with qRT-PCR (**Figure 8**). All DE genes followed the same expression trend with both RNA-seq and qRT-PCR except *USP28* which had up-regulated expression by qRT-PCR analysis and down-regulated expression with RNA-seq analysis.

## Discussion

### MAP Infection Throughout the Small Intestine of Cows With Subclinical JD

Animals selected for this study were serially tested during a period up to four years, using both serological- and fecal-based tests to identify cows with chronic MAP infection. The sensitivity of diagnostic tests from once-collected samples (cross-sectional studies) is low, in the order of 40-60% for blood ELISA (38). For this reason, we carefully selected negative and infected animals over a long period by combining faecal and serological detection tests. To ensure that animals were at the sub-clinical stage of the disease and also undergoing chronic infection, we recruited animals at 24 months of age and perform testing twice yearly. Furthermore, animals that advanced to the clinical stage of the infection, which were immediately culled, were not sampled. The phenotype designation based on the two tests was then further validated by qPCR to detect MAP-specific DNA and acid-fast staining in intestinal and lymph node tissue samples collected for transcriptional profiling. While these criteria established two distinct phenotypes, MAPinf and MAPneg cows, for comparative transcriptional profiling there may have been confounding factors. Aliquots of tissue that were used for the transcriptome analysis and the f57 PCR/acid fast-staining analyses are not the same although they were sliced from the same intestinal segment of the animal. In addition, the onset, and thus duration, of MAP infection was unknown for MAPinf animals. These are factors that might influence the substantial variation in MAP copy number/g of tissue among individuals as well as the results (**Table 1**).

It is difficult to determine if the transcriptome reflects immune protection or progression to JD. The primary value of the present study is that we identified naturally MAP-infected cows and age-matched controls to characterize mucosal transcriptome which reflects the response to MAP infection at two distinct, and prominent, anatomical sites in the small intestine. This is the first study to compare the transcriptomes of IL and JE, and ILLN and JELN in mature, naturally MAP-infected cows. Transcriptional profiles in jejunal and ileal intestinal mucosa of MAPinf cows, and in the associated draining mesenteric lymph nodes, reveal unique regional- and tissue-specific responses to MAP infection in subclinical cows. Common among the significant DE genes identified were numerous myeloid cell lineage, notably macrophages (*CD68*) and dendritic cells (*DCSTAMP*), and function-related genes in both IL and JE tissues from MAPinf cows.

### Tissue Differences in DE Genes During MAP Infection

Previous studies have shown regional differences among gastro-intestinal sites in terms of the number of expressed genes as well as DE genes at different stages of development in calves (39, 40). Also, more than 15,000 genes were expressed in each tissue which is similar to previous reports for bovine JE, IL and rumen tissues (40) (39). Moreover, Liang et al. (40) identified significant transcriptome differences between IL and JE of neonatal calves but these tissue differences did not persist in mature animals. Our results show substantial differential tissue responses to MAP infection as exemplified by the DE genes, enriched GO terms and pathways. More DE genes were detected in the IL (585 DE genes, 74% up-regulated) followed by JE (218 DE genes, 62% up-regulated) and JELN (205 DE genes, 58% up-regulated), while fewer genes were DE in the ILLN (117, 48% up-regulated) (**Figure 1A**) of MAPinf cows. This trend was reflected in pathways analysis and for enriched BP GO terms (**Figures 1B, C**). The greater host response to MAP in the IL *versus* the JE is somewhat surprising when considered in the context of mucosal immune defences in the bovine small intestine. In mature animals, it is presumed that the immune compartments (intraepithelium and LP) in the JE and IL are populated by similar immune effector cells. Prior to sexual maturity, the IL is occupied by a continuous PP that occupies two-thirds of the intestinal circumference. This PP is a primary lymphoid tissue functioning as a site of B cell development. Consistent with previous observations, this PP involutes following sexual maturity. Marked regional differences in response to MAP infection have been reported for the continuous PP in the IL when compared to discrete PPs in the JE of young calves (20, 41). Our study suggests regional-differences to MAP infection also occur in the intestinal tissue at these distinct anatomical sites in mature animals. The contribution of the local tissue response and the associated draining mesenteric lymph node to these differences remains to be determined. According to the results of DE genes, GO and pathways analyses, the ILLN displayed the least changes in transcriptome when compared to the JELN and the other tissues. Therefore, it appears the ILLN immune response might have been dampened by MAP or the ILLN may not have contributed substantially to the IL response to MAP infection. We detected more common DE genes between IL and JE (82 genes) and between the mesenteric lymph nodes (32 genes) than between intestinal tissue and lymph nodes (from 4 to 26 genes) (**Figure 1A**), thus confirming the anatomical and functional differences between these tissues. The lack of similarity between intestinal tissue and lymph nodes may, however, be influenced by selective recruitment of immune cells to the intestinal tissue. The abundance of DE genes in the IL may also be consistent with this region of the small intestine being an important site for the uptake and persistence of MAP infection (42). The robust host response to MAP infection observed in the IL raises important questions whether these responses contribute to control of infection or reflect immune evasion by MAP.

### The Altered Immune State in IL During Subclinical MAP Infection

Transcriptome analyses revealed that subclinical, persistent MAP infection alters a diverse range of immune responses, whereby primarily, immune response genes and disease/immune response associated GO terms and pathways were significantly enriched in the IL followed by JE, JELN and lastly, ILLN tissues. These data demonstrate that the immune response was more prominent in the IL of subclinical JD cows followed by the JE and JELN while being less active in the ILLN. Differences in the immune response by the IL and JE was further demonstrated by the identification of 65 DE genes, including genes of the HOX family (**Table 4**), when IL MAPinf was compared with JE MAPinf tissues. The HOX genes mostly function as transcriptional regulators so we performed Ki-67 staining to confirm if the degree of cell activation in the two tissues followed the pattern of HOX genes differential expression. Interestingly, Ki-67 staining revealed distinct or unique patterns of activated cells in each tissue, suggesting regional differences in host response to MAP-infection. It remains to be determined whether these Ki-67 stained cells represent similar immune cell subsets that are differentially organized in the IL and JE in response to MAP infection, or whether their spatial organization suggests they are different cell populations or possibly different subsets of either lymphoid or myeloid cells. Further work is warranted to identify which immune cells up-regulate HOX genes expression in the ileum when compared to the JE, and whether increased Ki-67 staining is directly linked to HOX genes up-regulation in these cells.

The IL has been regarded as the primary point of MAP invasion (10, 43, 44). Facciuolo et al., however, demonstrated that MAP is sampled equally by the continuous PP in the IL and the discrete PP in the JE of young calves (20). IHC examination of the intestinal tissues from MAPinf cows revealed more abundant CD172a staining in the LP and submucosa of IL and JE (highest in IL), followed by ILLN and JELN, when compared to MAPneg cows. Moreover, Ki-67 staining revealed abundance of activating cells distributed throughout the LP where activation of CD172a myeloid cells was also abundant in MAPinf IL tissues whereas in MAPinf JE tissues, proliferating cells were localized at the margins of areas staining intensely for CD172a (**Figure 6**). Increased CD172a staining suggest a greater abundance of myeloid cells in the tissues collected from MAPinf cows, especially in the IL, which is consistent with earlier observations of an increase in macrophages in the IL of MAP infected cattle (45, 46). This result is consistent with CD68, dendritic cell antigen *DCSTAMPI*, and CD172a myeloid cells playing a key role in facilitating MAP replication and the progression to JD. Moreover, IHC results showed increased CD3 T cell staining in the IL and JE LP and epithelium of MAPinf cows when compared to MAPneg cows (**Figure 6**). Greater staining of CD3 in the IL and JE is consistent with an activated immune state in these tissues. The higher activated immune state of the IL *versus* other tissues in subclinical cows is, however, somewhat surprising. This observation in older infected animals is very different from that reported by Facciuolo et al. for MAP infection in animals at 12 months of age (20). In this study (20), there were more extensive transcriptomic changes in the discrete or JE PPs *versus* continuous or IL PPs and these transcriptomic changes were associated with control of MAP infection in the JE region. Differences were also observed in the lymph node. However, CD3 staining was less abundant in ILLN and JELN tissues from MAPinf cows when compared to MAPneg cows (**Figure 7**). In addition, the presence of macrophages and dendritic cells markers (*CD68*, *DCSTAMP*) was absent in the ILLN in contrast to JELN, while it was increased for both IL and JE intestinal tissues. This suggests that antigen presenting cells migrate to a lesser extent to ILLN. Thus, the current study reveals substantial changes in host responses can occur in the IL when MAP infection persists beyond one year of age.

Further evidence for an activated state in the IL was apparent in the number of highly regulated genes (**Table 2**) as well as DE genes common to four or more enriched pathways (**Supplementary Table S4F**) with immune related functions. Some of these genes have previously been associated with either MAP infection in cattle or CD in humans (13, 17, 19, 47–53). Regardless of the bovine intestinal tissue sampled, the *CD14* (1.9 to 3.3 L2FC), and *LOC616364* (2.4 to 4.5 L2FC) genes were up-regulated while *ENSBTAG00000027033* was down-regulated (-2.2 to -4.8 L2FC) in response to MAP infection. These genes may be important signaling molecules during MAP infection. *CD14* is expressed by myeloid cells and the protein functions as a cell membrane receptor and soluble receptor for recognition of bacterial components. CD14 also interacts with TLR4 to induce NF-κB activation and cytokine expression (33, 54). Its enhanced expression in MAPinf tissues reflects a higher concentration of myeloid cells responding to the presence of MAP (demonstrated by IHC results) or possibly increased recruitment of monocytes (CD14+) in transition to becoming macrophages (CD14-, CD68+). Moreover, 24 additional members of the complement of differentiation gene family (e.g. *CD4, CD38, CD40, CD44, CD48*, etc.) were also DE (mostly up-regulated) in this study. *ENSBTAG00000048135*, a novel gene with unknown function in cattle, is a homologue of human *IGHG* gene. The *IGHG* gene family influence the innate immune function of IgG molecules and B-cells (53) and are associated with human tuberculosis (55) and JD in *Camelus dromedarius* (56). Another 73 novel genes (**Tables 2**, **3** and **Supplementary Tables S3A‒D**) including 8 (e.g. *ENSBTAG00000001595*, *ENSBTAG00000038067*, *ENSBTAG00000047363*, etc.) which increased more than four folds in one or more tissues and 12 (e.g. *ENSBTAG00000037578*, *ENSBTAG00000027033*, *ENSBTAG00000021407*, etc.) which decreased more than three folds in one or more tissues of MAPinf cows, suggest important functions during MAP infection and merit further investigations to understand their roles in JD progression. *LOC616364*, a homologue of human *CCL3* and also known as MIP-1α modulate inflammatory responses by binding to the receptors *CCR1*(up-regulated in JE and IL in this study), *CCR4* and *CCR5*.

The cumulative function of numerous individual genes contributing to diverse adaptive immune responses in the IL was evident through the major networks of gene interaction detected in this tissue (**Figure 4**) and the enriched pathways and GO terms. The Chemokine signaling pathway, Cytokine-cytokine receptor interaction and Phagosome pathway are amongst the most enriched pathways in the IL. The Phagosome pathway plays a central role in tissue remodelling, inflammation and defense against pathogens. Its enrichment with a high number of up-regulated DE genes of the adaptive and innate immune systems suggests a role in the response to a persistent MAP infection or may reflect increased survival of MAP infected macrophages and host responses to eliminate these cells. *CD14* and *CYBB* were among the DE genes of this pathway in the IL and also common to JELN. Many chemokine receptors were enriched in the Chemokine signaling pathway and Cytokine-cytokine receptor pathways in the IL. Many of these chemokine receptors (*CCL19, CCL4, CCR1, CCR7, CXCL11, CXCL13, CXCL16, CXCL8, CXCL9, CXCR3, CXCR4, CXCR5*, *LOC529196* **(**C-C chemokine receptor type 1-like), *LOC616364* **(**C-C motif chemokine 3) and *XCR1*) have been reported as DE during MAP infection in cattle (13, 19, 20). Moreover, chemokine receptors formed one of three major gene network clusters (**Figure 4**) in the IL supporting important roles for the chemokine signalling pathway and these molecules during MAP infection.

Other interesting pathways enriched for IL DE genes including TLR signaling pathway (also enriched in JELN), NF-κB signaling pathway (also enriched in ILLN), Antigen processing and presentation pathway, Leukocyte transendothelial migration pathway (also enriched in JELN) and NOD-like receptor signaling pathway support roles during MAP infection. Of the 11 bovine TLRs, *TLR2* and *TLR4* were significantly up-regulated in the JELN and IL, respectively and *TLR2* has been associated with a suppressed immune defense against MAP (57, 58). Meanwhile, the importance of NF-κB signaling pathway was seen through its potential activated state (enriched with up-regulated genes, n=13) in the IL but potentially diminished response (enriched with both up- and down-regulated genes, n=5) in the ILLN. Continual antigen presentation and recruitment of immune cells to the site of infection as a result of persistent MAP infection and continuous reinfection through dying macrophages is consistent with significant enrichment of Antigen processing and presentation pathway in the IL and Leukocyte transendothelial migration pathway in the IL and JELN. All the enriched genes (n=11) in the Leukocyte transendothelial migration pathway were significantly up-regulated in the IL. In particular, up-regulation of *CYBB* suggest an active role for leucocytes in the containment of MAP in infected cows. Leukocyte transendothelial migration pathway and other immune pathways (e.g. NOD-like receptor signalling, Lysosome and IL-17 signaling pathway) enriched in subclinical MAP infected cows in this study, have been reported as enriched in Holstein cows with clinical JD (16). The Antigen processing and presentation pathway and associated genes (*BOLA-DOA, CALR, CD4, CD74, CIITA, CTSS, IFI30, LGMN, LOC788634*) were up-regulated in the IL and many of these genes have been previously reported as up-regulated during early MAP infection in 2 to 4 months old calves (59). Therefore, enrichment of the Antigen processing and presentation pathway and related GO terms in the affected tissues, indicates that MAP infection is recognized by the adaptive immune system of cow small intestine. The NOD-like receptor signaling pathway was enriched with 25 DE genes in the IL, including *NOD1* and *NOD2*. Several studies have shown that NOD-like receptors are genetically associated with JD susceptibility (60, 61). It should be noted that, some disease pathways activated in this study are caused by pathogens or factors that subvert the host immune system by various mechanisms to promote their long term survival (e.g. Leishmaniasis (IL, and JELN), Legionellosis (IL and JELN), Toxoplasmosis (IL), Epstein-Barr virus infection (IL), Chagas disease or American trypanosomiasis (IL and JELN) and Inflammatory bowel disease (IL), etc.).

### MAP Elicits an Immune Response in JE

Our data show that MAP infection elicits pathogen-specific immune responses in the JE of mature cows. This is consistent with a recent report that MAP establishes persistent infections in the discrete PPs of the JE as well as the continuous PP of the IL (20). Furthermore, there were marked differences in host immune responses and control of MAP infection in these two regions of the small intestine (20). In our study, we examined intestinal tissue outside PPs and confirmed that JE intestine is a site for persistent MAP infection. This was confirmed by the presence of acid fast bacteria, qPCR detection of MAP-specific DNA, and transcriptome results. Gene expression data revealed a host immune response to MAP that included numerous highly expressed genes (e.g. *ENSBTAG00000027033*, *CD163*, *CTGF*, *LOC618463*, *DCSTAMP, CLEC4E, ENSBTAG00000047284* and *ENSBTAG00000018189*, *etc.)* and genes enriched in two or more KEGG pathways (e.g. *LOC615045, PLA2G2A, PLA2G5, CD14, CBR1, COL4A6, EIF4EBP1, LOC518526, MAP2K1, NOS2*, and *RAB7B*) (**Table 2** and **Supplementary Table S5C**), many of which are involved in immune responses, thereby suggesting roles during host response to MAP infection.

When compared to IL, only two (Ether lipid metabolism and Alpha-Linolenic acid metabolism) or one (Acute myeloid leukemia) of the seven pathways uniquely enriched among the JE DE genes had metabolic or disease related functions and further testifies to the differences in JE and IL responses to MAP infection. The activated immune/disease BP GO terms (about 59% [n=41]) and pathways (n=3) by DE genes in the JE is a clear indication that this tissue mounted an immune response to the presence of MAP, albeit not to the same extent as the IL. It remains to be determined whether this muted immune response reflects a more or less protective immune response.

### MAP Dampens Immune Processes in the ILLN During Subclinical Infection

In this study, the presence of MAP in the lymphoid tissues was confirmed by acid fast staining and qPCR detection of the *f57* single-copy DNA element unique to MAP (25). Generally, the JELN when compared to the ILLN was in an activated immune state with 36% of its 33 enriched BP GO terms and 13 of its 20 enriched KEGG pathways having immune/disease related functions (**Supplementary Tables S7A, B** and **Figure 5**). By contrast, only 10 BP GO terms and five (out of the seven) enriched KEGG pathways with disease/immune related functions were detected in the ILLN. The ILLN BP GO terms are related to B cell functions, lymphocyte activities and movement of immune cells. This data is consistent with the ILLN being the main site of B and T cell activation, supporting the constant movement of cells in and out of the ILLN in response to a continued MAP presence in macrophages and the IL environment. These data also demonstrate that persistent MAP infection may have compromised ILLN function with the most down-regulated DE genes (52.14%) recorded in the ILLN *versus* IL (26.32%), JE (38.07%) and JELN (42.44%). Three of the seven ILLN pathways were enriched for with mostly down-regulated genes including IL-17 signaling pathway and Melanoma. IL-17 signaling pathway and NF-*κ*B signaling pathway (common to IL and ILLN) are known to play significant roles in MAP and other mycobacterial infections. Interestingly, we observed in a previous study that differential regulation of cytokine production and T helper cells by IL-17A and IL-17F pathway in macrophages showed strong regulation of immunity-related genes during MAP infection (19). This observation is supported by the fact that CD3 (T cell marker) staining was markedly reduced in the ILLN of MAPinf cows. It has been shown that lymph nodes draining the mouse small intestine and colon are immunologically distinct and anatomically different and that dendritic cells that migrate to the draining intestinal lymph nodes are immunologically and anatomically separate (62). The dendritic antigenic marker *DCSTAMP* was absent in ILLN whereas it was found to be highly expressed (>7 log2FC) in JELN (**Supplementary Tables S3C, D**), which supports a regional difference associated with the migration of antigenic cells. If similar regional differences exist in the small intestine of cattle, this may explain the observed differential responses by ILLN and JELN to MAP infection in this study. Alternatively, there may be significant differences in MAP exposure at these sites or the immune inductive function of discrete PP in the JE versus the absence of PP in the IL may also contribute to these differences.

Amongst the ILLN pathways, Rheumatoid pathway and IL-17 signaling pathway ranked first. They are both enriched with mostly down-regulated genes in the ILLN (e.g., *CCL2*, *CCL20*, *FOS, IL17RB*) (**Supplementary Table S6B**). The IL-17 signalling pathway functions by maintaining intestinal barrier integrity, which is key to host protective capacity or immunopathology (63). Although IL-17 signaling pathway is a modest activator of signaling compared to other inflammatory stimuli, its capacity to synergize with other inflammatory signals, notably NF-κB, C/EBPβ, C/EBPδ and MAPK pathways, makes it a vital inflammatory effector (63). In MAPinf cows, this IL-17 signaling pathway is dampened in the ILLN and thus, is not working synergistically with the regional IL tissue which has a marked pro-inflammatory state. This dampened state in the ILLN is supported by the down-regulation of the transcription factor FOS, a component of a master transcriptional regulator of cells. Amongst the enriched genes in this pathways, *CCL2* (*MCP1*), is among the most down-regulated genes in ILLN (L2FC=-2.36, FDR = 2.92E-05) (**Supplementary Table S3C**). CCL2 and *CCL20* L2FC = -2.36 FDR = 2.92E-05 and L2FC = -2.09, FDR = 0.0142, respectively were reduced in the ILLN of MAPinf compared to MAPneg cows, and their involvement in chemotaxis (64, 65) might explain the absence of the dendritic antigenic marker *DCSTAMP* and the *CD68* markers in the ILLN tissue (**Supplementary Table S3C**). In macrophages from JD (MAPinf) cows, we also observed a reduction of *FOS* and its enrichment in the rheumatoid arthritis pathway, suggesting a role of this master transcriptional regulator in immunoregulatory and inflammatory processes (19). An additional master transcriptional regulator, the NF-κB signalling pathway, was dampened in the ILLN. The canonical NF-κB signalling pathway is triggered by numerous external stimuli such as TNF-alpha, TLR2, TLR4, and also through intracellular sensor such as NOD. The enriched genes (*BIRC3, CD40LG, LOC786717*) out of five in the NF-κB signaling pathway (**Supplementary Table S6B**) were down-regulated in the ILLN (**Supplementary Table S3C**) while *CD14* and *GADD45A* were up-regulated. Taken together, the detection of the most down-regulated genes (52.14%) and the enrichment of few immune/disease related pathways (n=5), including those enriched with mostly down-regulated genes (IL-17 signaling pathway, Malaria and Rheumatoid arthritis) in the ILLN potentially dampens their activities and could be one of the mechanisms used by MAP to subvert host responses and promote its long-term survival.

### Metabolic Processes Impacted by MAP in the Small Intestinal Tissues of Subclinical JD Cows

In this study, animals were in the subclinical phase of MAP infection and our data shows evidence of a MAP effect on metabolic processes. Notably, about 19%, 10%, and 24% of enriched BP GO terms in the IL, JE, and JELN, respectively are related to various metabolic processes, including lipid storage, lipid metabolic process, and regulation of lipid transport (**Supplementary Tables S4A**, **S5A**, and **S7A**), and in ILLN and JELN, Lipid and atherosclerosis (**Supplementary Tables S6A** and **S7A**). The impact of MAP on lipid homeostasis is particularly interesting because we have previously observed that macrophages from MAPinf cows (JD positive) accumulated lipid droplets (19). The accumulation in MAPinf macrophages was similar to the response of macrophages from healthy (MAPneg) cows exposed to MAP, and, interestingly, involved also Atherosclerosis Signaling pathway and HIF-1α Signaling pathway [Supplementary Table S9 in Ariel et al. (19)]. The HIF-1α Signaling pathway is crucial in the control of mycobacterial infection. HIF-1α regulates a metabolic transition to aerobic glycolysis which supports the inflammatory response of the host (66). However, MAP like other pathogenic mycobacteria relies on host lipids as carbohydrate source of energy. As a subversion strategy, MAP, like *Mycobacterium tuberculosis*, persistence inside host cells relies on metabolic adaptation, like the accumulation of lipid bodies in the so-called foamy macrophages (19, 67).

Several other metabolic-related pathways were also enriched by the DE genes, including Linoleic acid metabolism, Arachidonic acid metabolism, Fat digestion and absorption and Folate biosynthesis that were common to IL and ILLN (**Supplementary Tables S4B** and **S6B**). Five pathways enriched with down-regulated genes were identified in the IL (Vitamin digestion and absorption, Linoleic acid metabolism, Retinol metabolism, Arachidonic acid metabolism, Tryptophan metabolism, Fat digestion and absorption, and Folate biosynthesis) or one (Arachidonic acid metabolism) enriched with down-regulated genes in the JE. During the immediate response to mycobacterial infection, the TLR-mediated signaling cascade releases arachidonic acid by the action of phospholipases (68). Breakdown of arachidonic acid, an integral part of all cell membranes, leads to production of lipids that are metabolized notably by the Arachidonic acid metabolism pathway. The arachidonate 5-lipoxygenase (ALOX5), an important actor of this pathway, generates leukotrienes which are pro-inflammatory lipid mediators. The *ALOX5* gene was downregulated in JE from MAPinf cows. In our previous study, down-regulation of *ALOX5* was also observed in bovine macrophages exposed to MAP infection (19). We also identified in this previous study down-regulation of phospholipase genes, notably encoding LDL-associated phospholipase A2 genes. In the current study, *PLA2G2A* and *PLA2G5*, part of the Arachidonic acid metabolism pathway, were down-regulated in the JE (**Figure 3B** and **Supplementary Table S3B**). The lipid mediator leukotriene promotes inflammation. Downregulation of *ALOX5* and LDL-associated phospholipase A2 genes would be an efficient host strategy to control the chronic inflammation that a persistent MAP infection would induce in the JE tissue. These data are consistent with the IL and JE being the main site of digestion and absorption and provide evidence that MAP infection impacts metabolic processes, especially in the IL, JE and JELN during subclinical MAP infection. Moreover, these data indicate that animals were already adapting to changing intestinal morphology, energy shortages as indicated by some of the enriched pathways (NOD-like receptor signaling pathway, HIF-1 signaling pathways). The animals were followed over a one- to four-year period and the current data indicate that cows may have been approaching the clinical stage of infection. In support of our findings, several enriched metabolic pathways (Arginine and proline metabolism, Arachidonic acid metabolism, Linoleic acid metabolism, Fructose/mannose metabolism and Retinol metabolism) have been identified in the ileocecal valve of Holstein cattle with subclinical MAP infection (16). Another study identified three metabolic pathways (N-Glycan biosynthesis, Purine metabolism and One carbon pool by folate route) in the ileocecal valve of cows naturally infected with MAP (13). Studies on metabolic profiling of cattle experimentally infected with MAP indicate energy shortages, increased fat metabolism, increased protein turnover and altered concentrations of N-glycans, such as mannose (69), as well as increased serum levels of vitamin D binding protein precursor, transthyretin, retinol binding protein, and cathelicidin in infected cattle (70). It was proposed recently that *Mycobacterium tuberculosis* infection can manipulate the glycosylation machinery and the N-Glycoproteome of human macrophages (71).

Our data show that not only immune functions but also metabolism are important aspects of the host response to MAP infection. The importance of immunometabolism has been highlighted for many diseases including tuberculosis (72, 73). Therefore, cellular metabolism is considered an important immune regulator (74). After the incubation stage, MAP requires more nutrients to support its rapid growth within host cells and possibly perturb the cellular metabolism of immune cells to further facilitate its survival and persistence in the host. It is also possible, however, that the host may adapt to MAP presence and the rapid increase in MAP cells by altering gut metabolism to provide more nutrients for exhausted immune cells. Therefore, understanding the complex immunometabolism interactions during MAP infection is important for understanding the pathogenesis of JD. This implies that novel aspects of the pathogenesis and host immune responses to MAP infection could be tied to metabolic processes and thus require further attention.

## Conclusion

In summary, host adaptation to MAP infection varied greatly among local enteric microenvironments, displaying regional- and tissue-specific host responses to subclinical MAP infection. The IL displayed the highest number of DE genes and enriched immune/disease related GO terms and pathways. The JE tissue displayed measurable immune response to subclinical MAP infection as evidenced by acid fast staining, detection of MAP-specific DNA and gene expression, confirming that the JE is an important site of MAP infection. The JELN also responded to MAP presence but to a lesser extent when compared to IL and JE. The effects of MAP on metabolic pathways and BP GO terms was more pronounced in the IL and JE. The ILLN presented a dampened immune state characterized by the highest numbers of down-regulated immune/disease genes and only five immune pathways (including two enriched with only down-regulated genes and one enriched with mostly down-regulated genes), indicating that the ILLN may be in a dysfunctional state, responding minimally to MAP infection or responses were suppressed by the pathogen during a sustained subclinical MAP infection. Several dysregulated immune genes (e.g., those listed in **Tables 2** **‒4**) identified in this study could be potential biomarkers of MAP infection, and their specific roles in disease pathogenesis also merits further investigation. In addition, immuno-metabolism was identified as a potential novel aspect of the pathogenesis and host immune responses to MAP infection. Several novel genes associated with MAP infection also warrant further investigation to determine their role in MAP pathobiology. Finally, this study provides insight into the extensive transcriptomic changes underlying the host response to a persistent subclinical MAP infection in the bovine small intestine.

## Data Availability Statement

The datasets presented in this study can be found in online Repositories and in the article/**Supplementary Material**. The raw datasets generated for this study have been deposited in the NCBI Sequence Read Archive (SRA) under the BioProject ID PRJNA729129.

## Ethics Statement

The animal study was reviewed and approved by Animal Care and Ethics Committee of Agriculture and Agri-Food Canada.

## Author Contributions

The study was designed by EI-A, NB, and PG. NB performed the longitudinal study of 16 herds, collected samples, performed the blood ELISA and fecal qPCR, and selected animals for necropsy. DND, MW, and P-LD performed the bioinformatics and pathways analyses. AF and P-LD performed the f57 qPCR analysis, AF analyzed the immunohistochemical slides/data. EI-A and DND drafted the manuscript. EI-A, NB, AF, and PG thoroughly revised the manuscript. EI-A, DND, PG, AF, and NB provided inputs in interpretation of the results. All authors revised and approved the final manuscript.

## Funding

This study was funded by Agriculture and Agri-Food Canada (Projects #J000079 and #J002223).

## Conflict of Interest

The authors declare that the research was conducted in the absence of any commercial or financial relationships that could be construed as a potential conflict of interest.

## Publisher’s Note

All claims expressed in this article are solely those of the authors and do not necessarily represent those of their affiliated organizations, or those of the publisher, the editors and the reviewers. Any product that may be evaluated in this article, or claim that may be made by its manufacturer, is not guaranteed or endorsed by the publisher.

## Supplementary Material

Supplementary Figure 1 Gene interaction networks between DE genes in the jejunum

Supplementary Figure 2 Gene interaction networks between DE genes in the **(A)** Ileal and **(B)** jejunal lymph nodes

Supplementary Figure 3 CD 14 gene expression in IL and JE tissues of MAP-infected cows compared with IL and JE tissues of MAP negative cows.

Supplementary Figure 4 Immunohistochemical staining of intestinal tissue and mesenteric lymph nodes for CD11c+cells. Representative tissue sections from a MAP-infected cow **(A, C, E)** and a MAP-negative cow **(B, D, F)** stained for the myeloid cell surface marker CD11c (brown stain).
