# Supplementary figures and images for: Regionally Distinct Immune and Metabolic Transcriptional Responses in the Bovine Small Intestine and Draining Lymph Nodes During a Subclinical Mycobacterium avium subsp. paratuberculosis Infection
Source: Front Immunol. 2021 Dec 15;12:760931. doi: 10.3389/fimmu.2021.760931 (PMC8714790; doi:10.3389/fimmu.2021.760931)

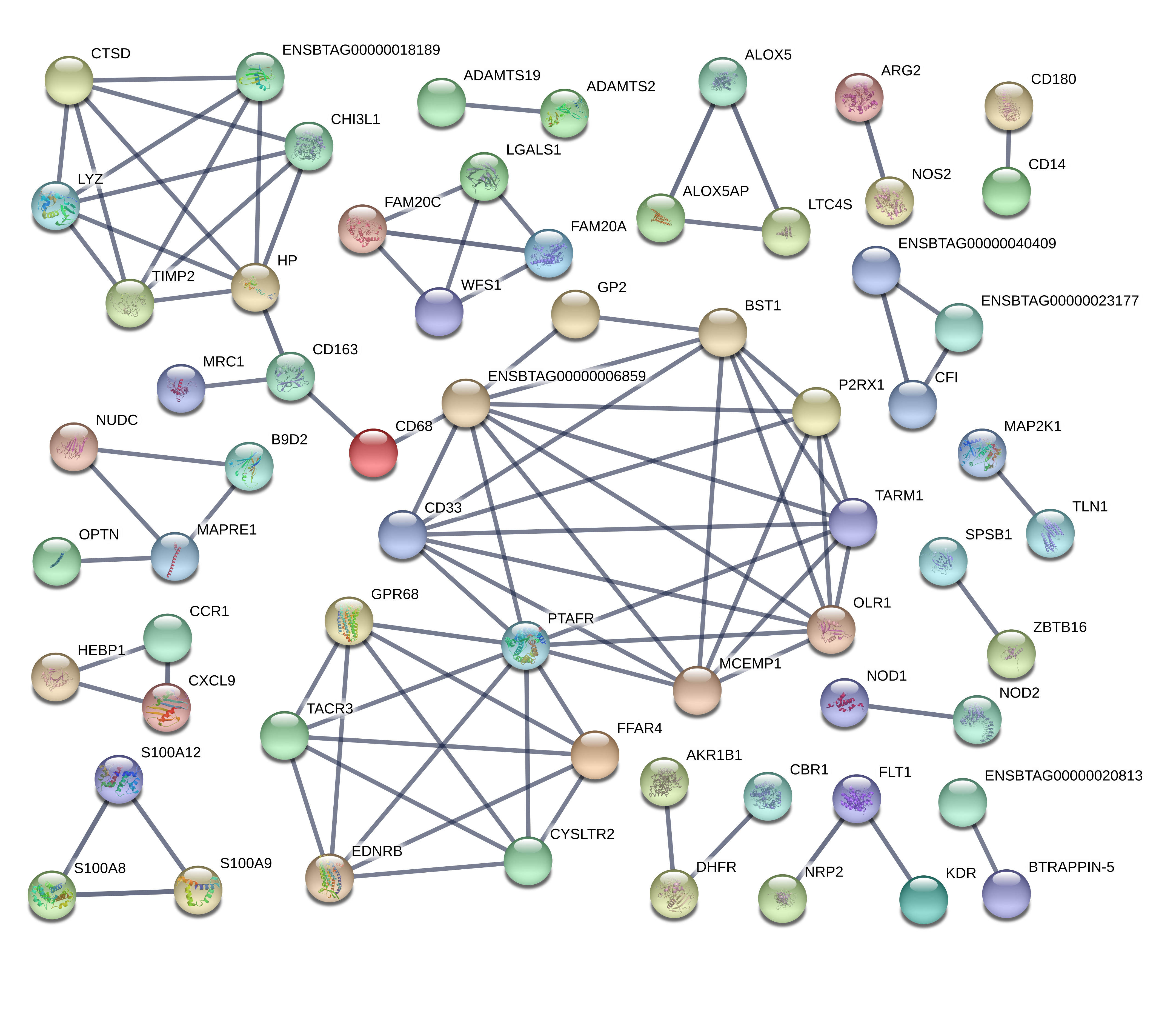

Supplement: Supplementary Figure 1 — Gene interaction networks between DE genes in the jejunum [file Image_1.jpeg]

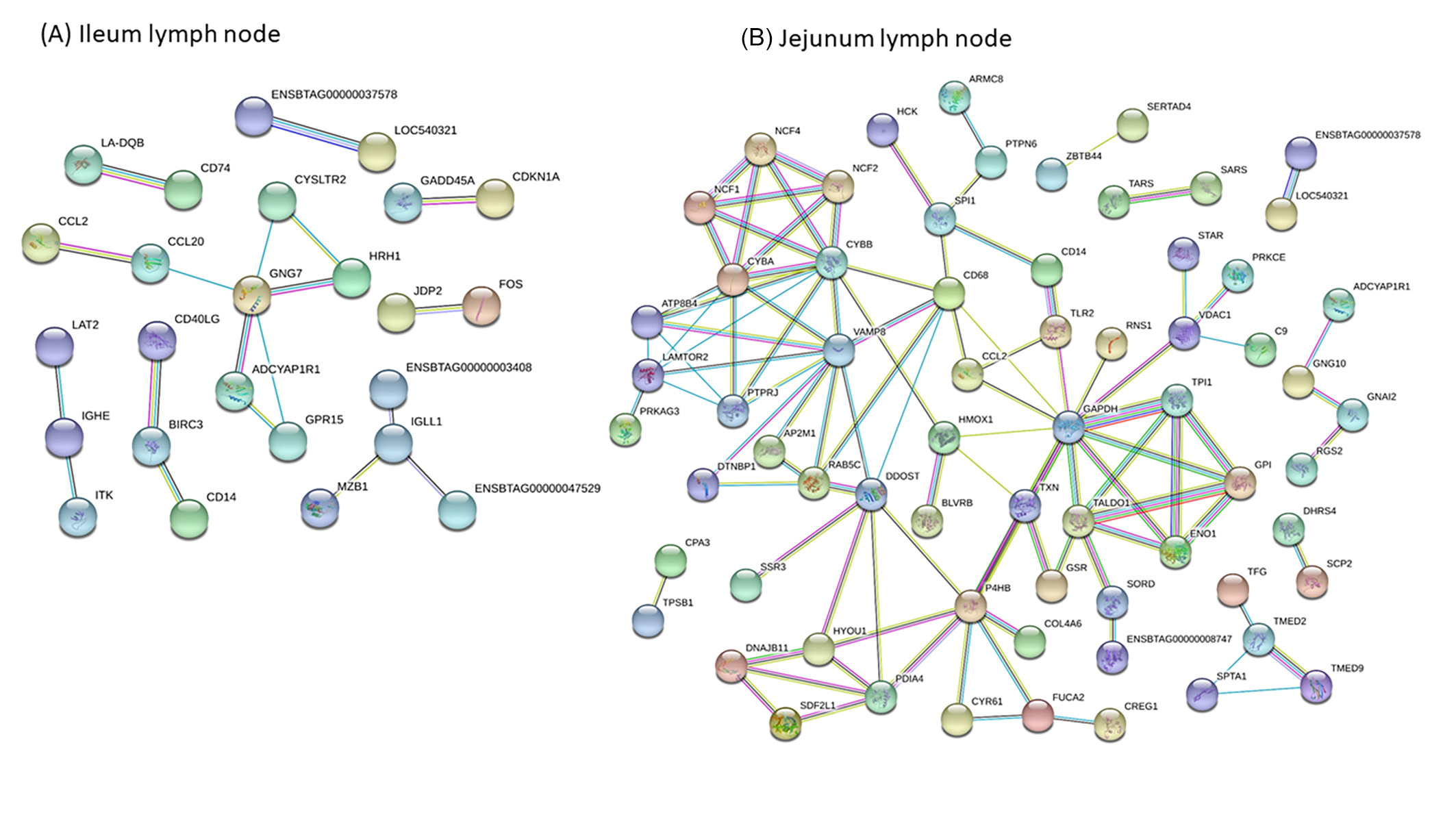

Supplement: Supplementary Figure 2 — Gene interaction networks between DE genes in the (A) Ileal and (B) jejunal lymph nodes [file Image_2.tif]

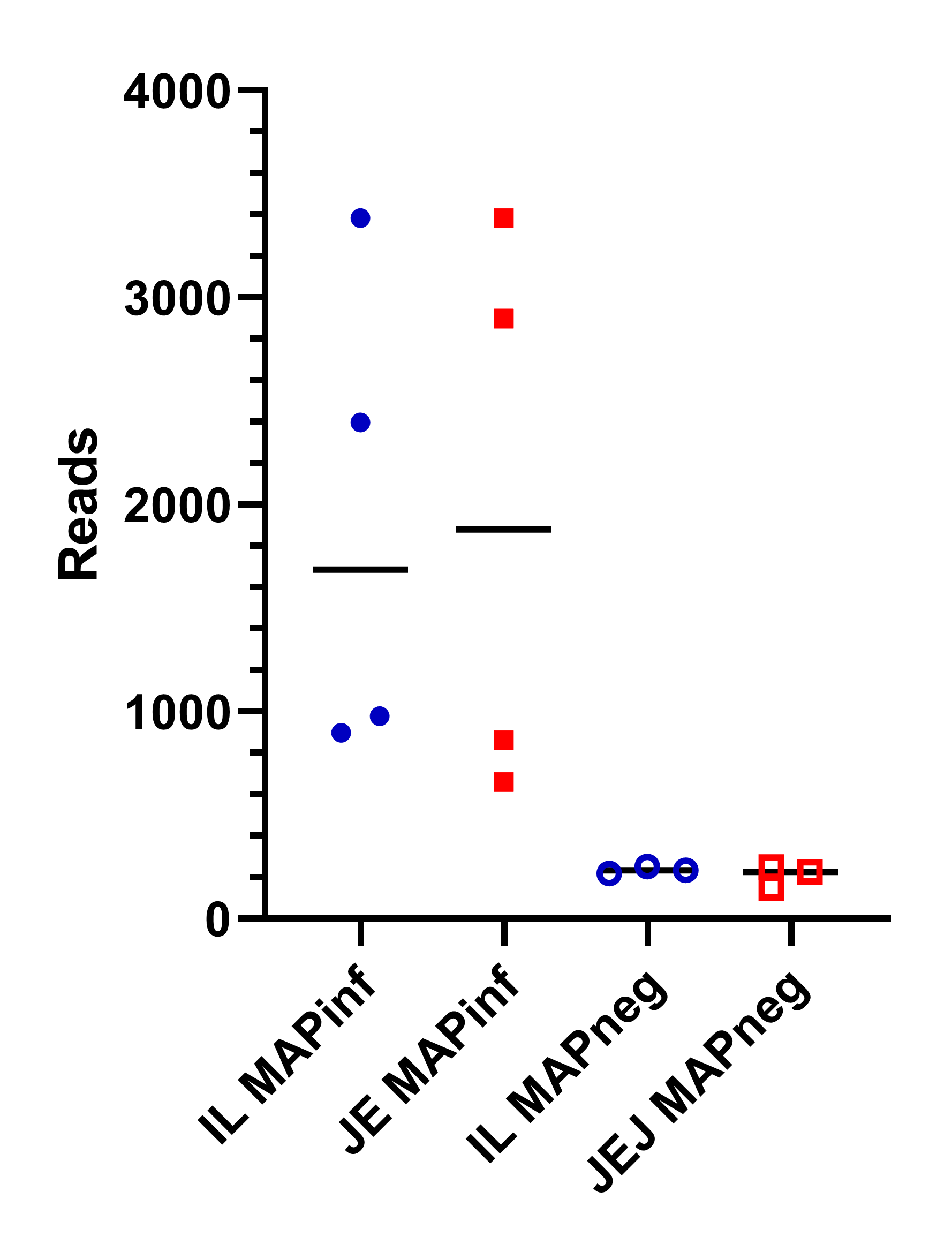

Supplement: Supplementary Figure 3 — CD 14 gene expression in IL and JE tissues of MAP-infected cows compared with IL and JE tissues of MAP negative cows. [file Image_3.tif]

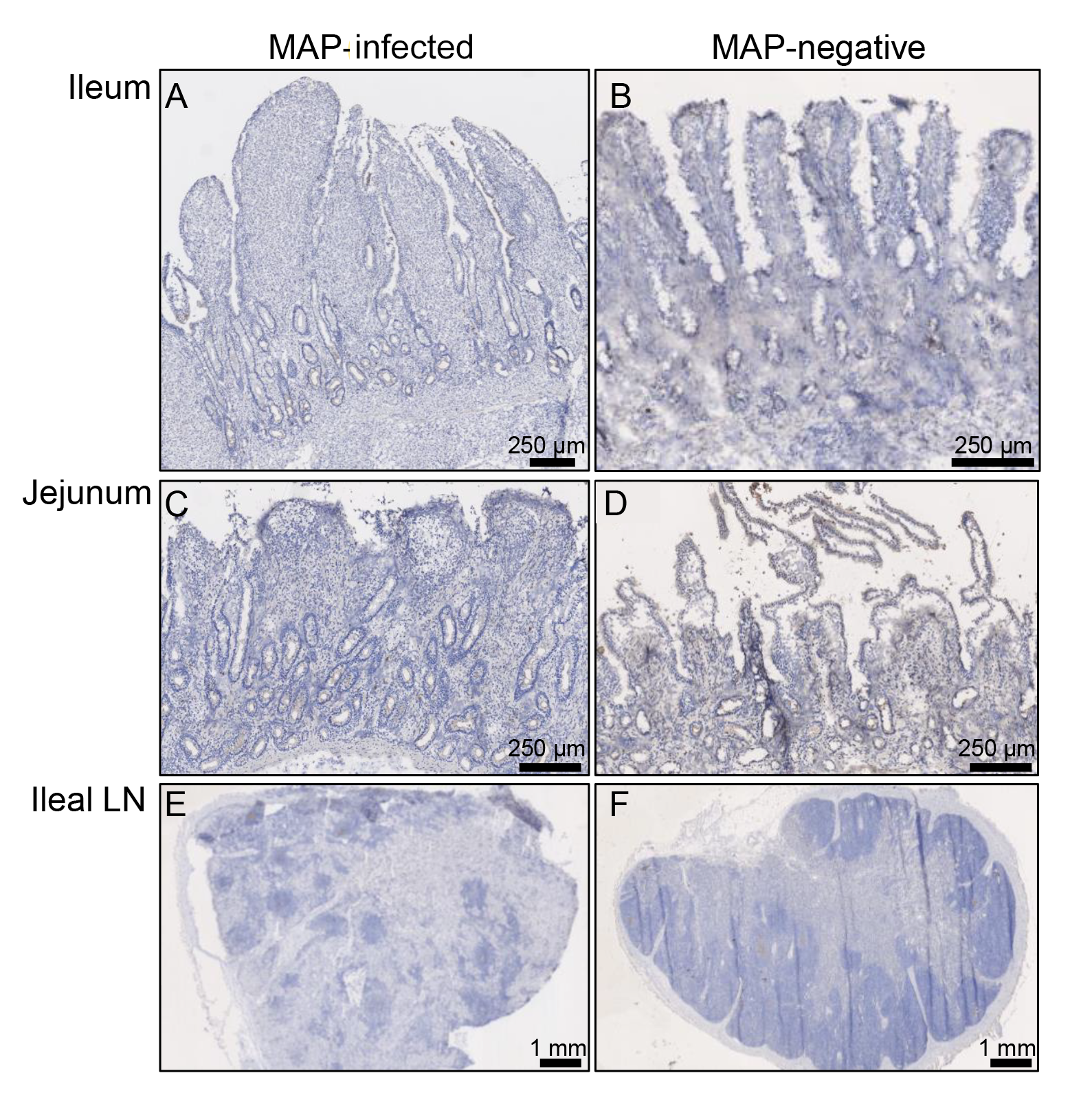

Supplement: Supplementary Figure 4 — Immunohistochemical staining of intestinal tissue and mesenteric lymph nodes for CD11c+cells. Representative tissue sections from a MAP-infected cow (A, C, E) and a MAP-negative cow (B, D, F) stained for the myeloid cell surface marker CD11c (brown stain). [file Image_4.tif]
