# Supplementary material for: Regionally Distinct Immune and Metabolic Transcriptional Responses in the Bovine Small Intestine and Draining Lymph Nodes During a Subclinical Mycobacterium avium subsp. paratuberculosis Infection
Source: Front Immunol. 2021 Dec 15;12:760931. doi: 10.3389/fimmu.2021.760931 (PMC8714790; doi:10.3389/fimmu.2021.760931)
Supplement: Supplementary file 5 [file Table_1.docx]

**Supplementary table**

**Table S1. Genes and primer sequences used in qPCR validation of RNA-sequencing data**

| Target gene | Description/Tissue | Accession number | Primer | Concentration (nM) | Sequence | Amplicon length |
| --- | --- | --- | --- | --- | --- | --- |
| RPS15 | Non DE/ Housekeeping | NM_001024541.2 | Forward | 300 | 5’-GAT CAT TCT ACC CGA GAT GGT G-3’ | 127 |
|  |  |  | Reverse | 300 | 5’-GGG CTT GTA AGT GAT GGA GAA-3’ |  |
| ATP5B | Non DE/ Housekeeping | NM_175796.3 | Forward | 300 | 5’-ATC ACC ACC ACC AAA AAG GGA-3’ | 108 |
|  |  |  | Reverse | 300 | 5’-AGC ATC CAA ATG GGC AAA GG-3’ |  |
| PGK1 | Non DE | NM_001034299.1 | Forward | 300 | 5’-ACA AGC TGA CTC TGG ACA AGC-3’ | 113 |
|  |  |  | Reverse | 300 | 5’-AGC CTT GAT CCT CTG GTT GTT-3’ |  |
| RPS9 | Non DE | NM_001101152.2 | Forward | 300 | 5’-TTT CCA GAG CGT TGG CTT AG-3’ | 114 |
|  |  |  | Reverse | 300 | 5’-GGA CTT CTC GAA GGG TCT CC-3’ |  |
| CD14 | Ileum lymph node | NM_174008.1 | Forward | 150 | 5’-TGA ACA TTG CCC AAG CAC AC-3’ | 101 |
|  |  |  | Reverse | 300 | 5’-GCC GAG ACT GGG ATT GTC AG-3’ |  |
| PTN | Ileum lymph node | NM_173955.1 | Forward | 300 | 5’-CAC CAG CGA CTT GGG TAC CTG-3’ | 116 |
|  |  |  | Reverse | 900 | 5’-CAG GTA CTG TGG AGT CTG CAT T-3’ |  |
|  |  |  | Reverse | 300 | 5’- ATT ACA AGG GCT GAA GGA AGG AA-3’ |  |
| NFYA | Jejunum lymph node | NM_001014956.1 | Forward | 300 | 5’-TCA GGC CAG CAA GTC CA-3’ | 100 |
|  |  |  | Reverse | 300 | 5’-TGA TGG GTT GGC CAG TTG ATG-3’ |  |
| HRSP12 | Jejunum lymph node | NM_001034208.2 | Forward | 300 | 5’-ATA ATC AGC ACC GCG AAA GC-3’ | 101 |
|  |  |  | Reverse | 300 | 5’-GGG TCC ATG CCT AGT TGT CC-3’ |  |
| SLC7A6 | Jejunum lymph node | NM_001076785.3 | Forward | 300 | 5’-CTG TCG CCC TGT CCT GTT TT-3’ | 113 |
|  |  |  | Reverse | 900 | 5’-GTG GAT CAT GGA CAG GAG GT-3’ |  |
| SNX10 | Jejunum lymph node | NM_001075375.1 | Forward | 300 | 5’-CGC CGG ATT GAT CGT GTT CT-3’ | 111 |
|  |  |  | Reverse | 300 | 5’-AGT GCC AGA AGT CCT CCT TC-3’ |  |
